# Suppressing the Hypoxia‐Adenosinergic Axis by a Tailored Nanoreactor for Enhanced Photothermal Immunotherapy

**DOI:** 10.1002/smsc.202300242

**Published:** 2024-02-05

**Authors:** Jingjing Gu, Jiao Chang, Shiyu Chen, Hui Zhi, Jiuyuan Sun, Weimin Yin, Tingting Zhang, Jie Zang, Yuge Zhao, Yiqiong Liu, Xiao Zheng, Leiyu Feng, Yongyong Li, Haiqing Dong

**Affiliations:** ^1^ Key Laboratory of Spine and Spinal Cord Injury Repair and Regeneration Ministry of Education Tongji Hospital The Institute for Biomedical Engineering & Nano Science School of Medicine Tongji University Shanghai 200092 P. R. China; ^2^ Shanghai Skin Disease Hospital The Institute for Biomedical Engineering & Nano Science School of Medicine Tongji University Shanghai 200092 P. R. China; ^3^ State Key Laboratory of Pollution Control and Resources Reuse School of Environmental Science and Engineering Tongji University Shanghai 200092 P. R. China

**Keywords:** adenosinergic axis, hypoxia relief, immunogenic cell death, immunosuppression, photothermal immunotherapy

## Abstract

Cell metabolite adenosine can induce extensive and persistent immunosuppression by binding to adenosine receptors on immune cells. Seriously, the hypoxia‐driven adenosinergic axis aggravates adenosine accumulation via dephosphorizing immune‐activating adenosine triphosphate (ATP) released during immunogenic cell death (ICD). Different from direct adenosine clearance or adenosine receptor blockade or directly using ecto‐enzyme (CD39/CD73) antagonist, it is hoped to use an innovative small science engineering to regulate the upstream hypoxia/HIF‐1α signal of the hypoxia‐adenosinergic axis, thereby reducing the immunosuppressive extracellular adenosine and enhancing ICD‐triggered antitumor immunity. PM@Mn is constructed by gradually integrating metformin and MnO_2_ on polydopamine (PDA) nanoparticles. PM@Mn can effectively suppress hypoxia‐adenosinergic axis via combining catalytic oxygen production with reduced endogenous oxygen consumption. Such motif of hypoxia relief suppresses the metabolism of ATP to adenosine via down‐regulating the expression of HIF‐1α, CD39, and CD73. Meanwhile, PDA in PM@Mn can induce local tumor ablation and trigger the “vaccine effect” of ICD under near‐infrared radiation. In a mouse breast cancer model with low immunogenicity, our strategy can effectively reduce adenosine accumulation, PM@Mn group exhibits 4.51‐fold cytotoxic T lymphocyte infiltration and tumor inhibition rate of 75.4%. This study provides a new strategy to advance ICD‐triggered antitumor immunity through supressing hypoxia‐adenosinergic axis.

## Graphical abstract

PM@Mn can effectively suppress hypoxiaadenosinergic axis via combining catalytic oxygen production with reduced endogenous oxygen consumption. Such motif of hypoxia relief suppresses the metabolism of ATP to adenosine via down‐regulating the expression of HIF‐1α, CD39, and CD73. Meanwhile, photothermal effect of PDA in PM@Mn can induce local tumor ablation and trigger the “vaccine effect” of ICD under near‐infrared radiation.

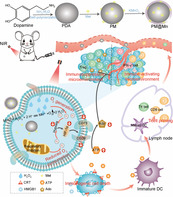

## 1 Introduction

Immunotherapy exhibits an unpleasant therapeutic effect on solid tumors, which is mainly ascribed to the low immunogenicity with barren killer T cells infiltration.^[^ ^1^ ^]^ Immunogenic cell death (ICD) can activate adaptive immunity by releasing tumor‐associated antigens and damage‐associated molecular patterns while locally damaging tumors.^[^ ^2^ ^]^ Thus, ICD induction is considered as an attractive strategy to enhance “cold tumor” immunogenicity and activate antitumor immunity. Nevertheless, innate negative regulatory mechanisms exist in the tumor microenvironment (TME) to prevent immune hyperactivation.^[^ ^3^ ^]^ For instance, adenosine triphosphate (ATP), a key biomarker for immune activation during ICD, is hydrolyzed rapidly to adenosine with the help of concomitant up‐regulated ectonucleotidases CD39 and CD73.[^3^, ^4^] Extracellular adenosine binds to A2A receptors (A2AR) distributed on the surface of various immune cells, which triggers the accumulation of intracellular cyclic adenosine monophosphate (cAMP) and then initiates the signaling cascades mediated by cAMP‐dependent protein kinase A, thereby inhibiting T cell effector functions and promoting immunosuppressive transcription via cAMP response elements.^[^ ^5^ ^]^ Sitkovsky team has revealed that the adenosine‐A2AR‐cAMP axis is an important physiological negative feedback mechanism in the regulation of inflammatory and immune responses.^[^ ^6^ ^]^ Adenosine, an over‐accumulated metabolite, is 17 times higher in TME than in normal tissue.^[^ ^7^ ^]^ Hence, regulation of adenosinergic axis (ATP‐CD39/CD73‐adenosine‐A2AR‐cAMP pathway) is particularly critical for enhancing ICD‐triggered antitumor immunity.

Currently, preclinical and clinical studies on the regulation of adenosinergic axis mostly focus on three aspects: 1) resisting adenosine generation via direct administration of small molecule antagonists or monoclonal antibodies targeting ectonucleotidase CD39 or CD73;^[^ ^8^ ^]^ 2) enhancing adenosine clearance via supplying adenosine deaminase,^[^ ^9^ ^]^ and 3) inhibiting the interaction between adenosine and its receptor via delivery of small molecule antagonists targeting adenosine 2A receptor.^[^ ^10^ ^]^ Strikingly, resisting adenosine generation may reverse adenosine‐mediated immunosuppression while upregulating the immune‐activating ATP,^[8b]^ appears to be more attractive than other strategies. Preclinical biochemical studies have established that tumor hypoxia stabilizes HIF‐1α, thus boosting transcription of ectonucleotidases CD39 and CD73, whose gene promoters contain hypoxia response elements.^[6a]^ Namely, hypoxia/HIF‐1α signaling are upstream of adenosine‐A2AR‐cAMP axis, and HIF‐1α–mediated accumulation of extracellular adenosine triggers cAMP‐mediated immunosuppression.^[^ ^11^ ^]^ Taken together, hypoxia‐adenosinergic immunosuppression was discovered and understood as an outcome of parallel studies of hypoxia‐HIF‐1α axis and adenosine receptors‐cAMP axis. HIF‐1α, involved in the regulation of hundreds of hypoxic homeostasis response genes^[^ ^12^ ^]^ and acts as an upstream mediator of the hypoxia‐adenosinergic pathway,[^11^, ^13^] has become an important target in tumor biology research. Studies established that systemic hyperoxic breathing relieved the tumor hypoxia, as well as downregulated the levels of HIF‐1α and downstream target proteins of HIF‐1α (e.g., CD39 and CD73) in the TME, thus decreasing levels of extracellular adenosine.^[5c]^ Although systemic hyperoxic breathing was shown to be a practicable method to suppress the hypoxia‐adenosinergic axis, there are obvious limitations for oxygen transport and delivery farther away from blood vessels due to irregular blood vessels within the tumor.^[^ ^14^ ^]^ Hence, different from the systemic hyperoxic breathing or competitive inhibition or blocking by small molecule antagonists or monoclonal antibodies,^[^ ^15^ ^]^ we hope to use an innovative small science engineering to regulate the upstream hypoxia/HIF‐1α signal of the hypoxia‐adenosinergic axis, thereby reducing the immunosuppressive extracellular adenosine and enhancing ICD‐triggered antitumor immunity.

Nanomedicine has been extensively explored to improve tumor hypoxia, including catalytic oxygen production, direct delivery of oxygen, improvement of blood perfusion, and inhibition of oxygen consumption, etc.^[^ ^16^ ^]^ Notably, current research is mostly devoted to sensitizing radiotherapy or photodynamic therapy itself. Since radiotherapy and photodynamic therapy are oxygen‐dependent therapies, local hypoxia will inevitably be aggravated in the actual treatment.^[^ ^17^ ^]^ Moreover, the strategies mentioned above face certain challenges, i.e., relatively limited endogenous hydrogen peroxide (H_2_O_2_) concentration in tumors that is insufficient to meet the needs of tumor catalytic therapy,^[^ ^18^ ^]^ premature oxygen (O_2_) leakage and limited effect of hypoxia relief.^[^ ^19^ ^]^ Summarily, efficient tumor oxygenation requires a comprehensive consideration of oxygen level and oxygen persistence, and perhaps the combination of exogenous and endogenous oxygenation can be imperative to display respective superiority and effectively ameliorate tumor hypoxia.

**Scheme 1:**
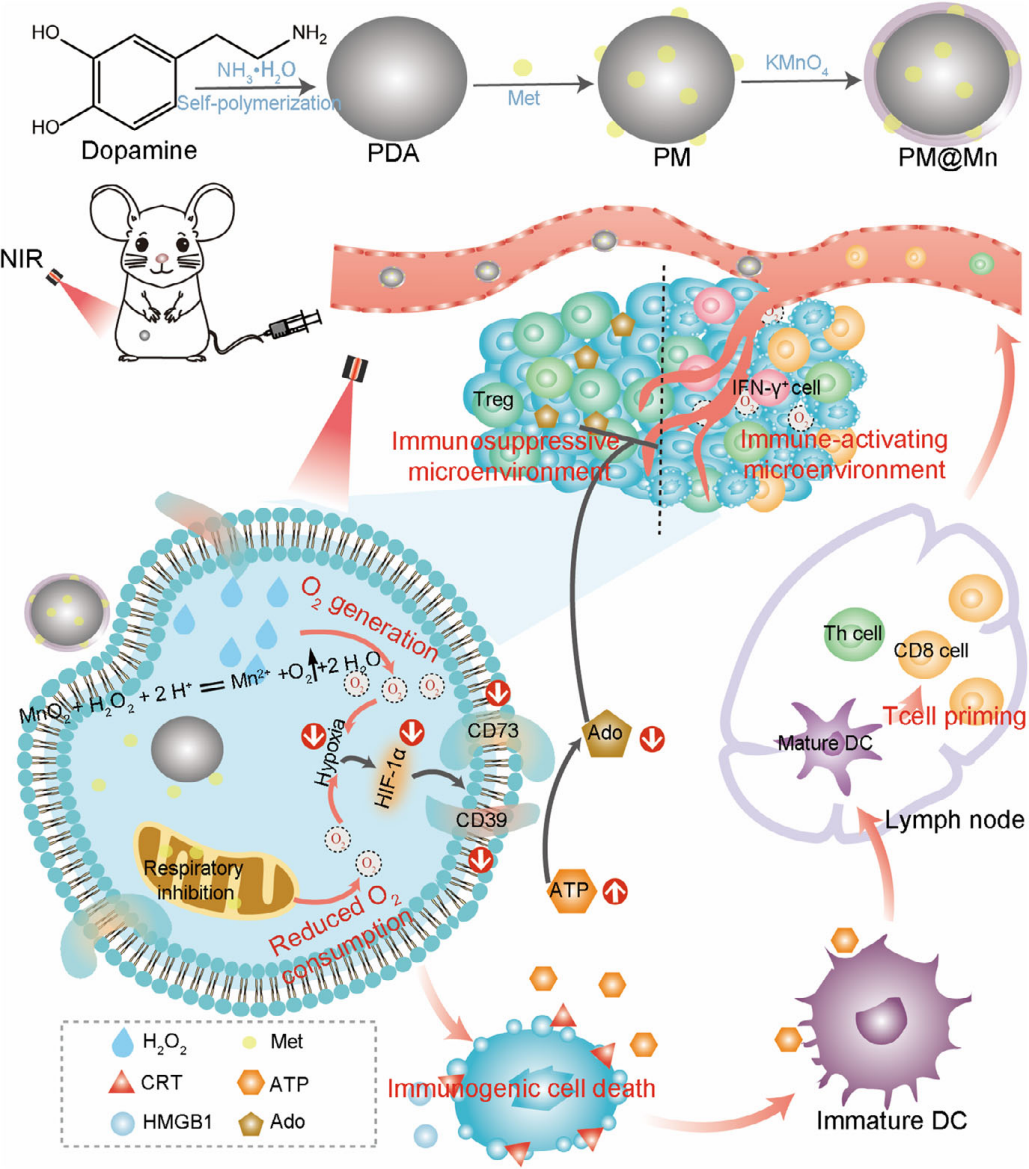
Schematic illustration of PM@Mn under near‐infrared radiation to remodel hypoxia‐adenosinergic axis for enhanced photothermal immunotherapy.

Photothermal therapy (PTT) has the advantages of minimal invasiveness, safety, and controllability,^[3b]^ and its antitumor effect does not depend on the oxygen level of tissues. To effectively improve the hypoxic microenvironment of solid tumors, reshape the HIF‐1α‐dependent adenosinergic axis, and then amplify the ICD‐triggered antitumor immunity, we took full advantage of the photothermal properties, carrier function and reducibility of polydopamine (PDA) to construct PM@Mn nanoreactor via loading the mitochondrial respiration inhibitor metformin (Met) and generating manganese dioxide (MnO_2_) in situ (**Scheme** **1**). Specifically, PM@Mn could induce local tumor ablation and trigger the “vaccine effect” of ICD under near‐infrared radiation (NIR). Meanwhile, it could effectively remodel hypoxia‐adenosinergic axis via combining catalytic oxygen production with reduced endogenous oxygen consumption, thereby with the expectation of suppressing the metabolism of ATP to adenosine. Even in a mouse breast cancer (4T1) model with low immunogenicity, our strategy still achieved robust immune activation and tumor suppression. This project enriches the research on the regulation of adenosine metabolism, and provides a new avenue for the regulation of tumor metabolism to amplify the ICD‐triggered antitumor immunity.

## 2 Results and Discussion

### 2.1 Construction and Characterization of PM@Mn

**Figure 1:**
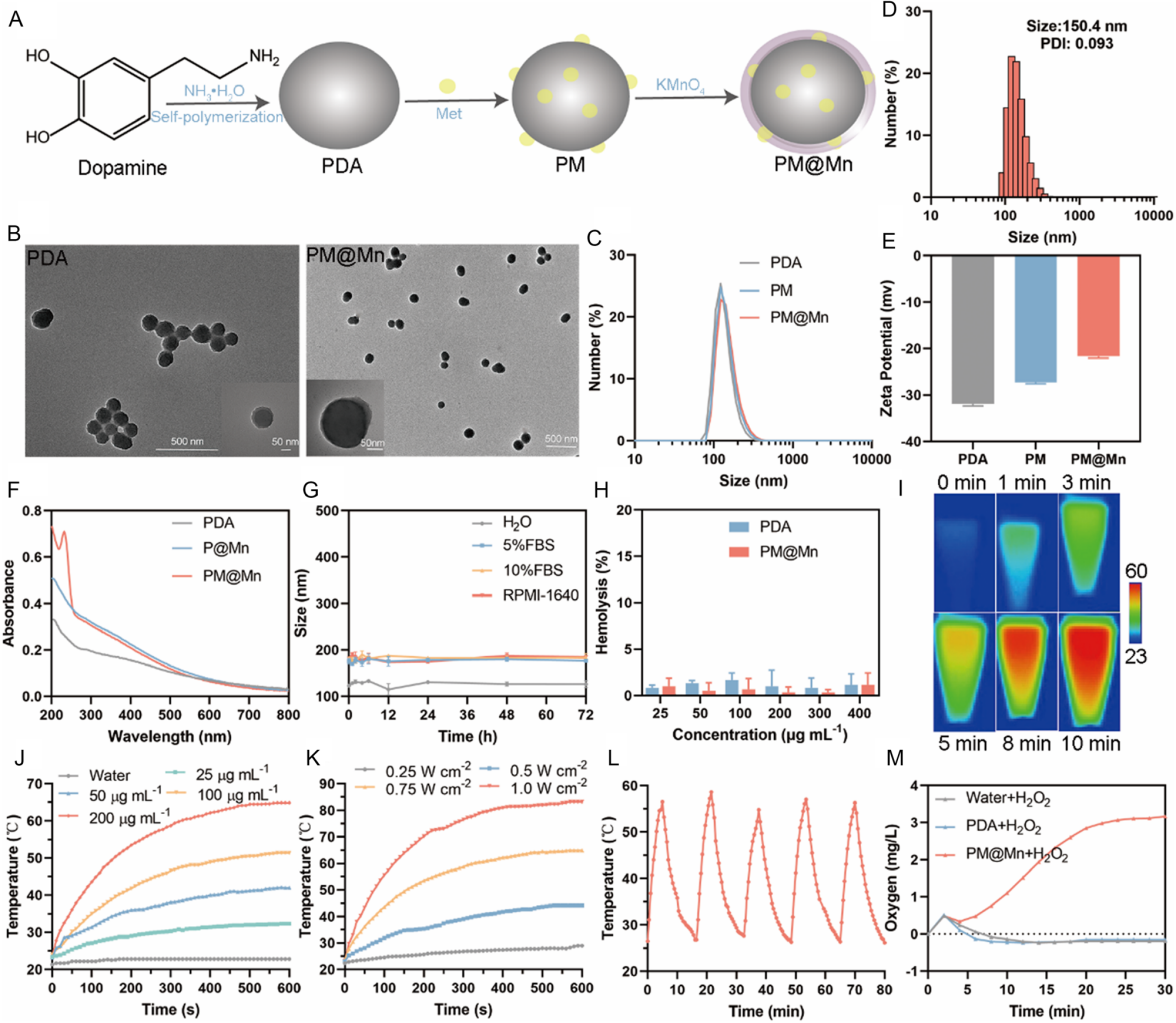
Construction and characterization of PM@Mn. A) Schematic illustration of the preparation process of PM@Mn. B) TEM images of PDA and PM@Mn under different fields of view. C) Particle size distribution of nanoparticles with different components. D) Particle size distribution and PDI of PM@Mn. E) Zeta potential of nanoparticles with different components. F) Ultraviolet‐visible absorption spectra of nanoparticles with different components. G) Particle size variations of PM@Mn in different dispersion media. H) Hemolysis rates of PDA and PM@Mn at different concentrations. I) Infrared thermal images of PM@Mn (200 μg mL^−1^) irradiated by an 808 nm laser (0.75 W cm^−2^). J) Temperature curves of PM@Mn with different concentrations. K) Temperature curves of PM@Mn under different irradiation power. L) The temperature‐cooling cycle curve of PM@Mn under irradiation (1 W cm^−2^, 5 min each time). M) Oxygen content changes of PDA and PM@Mn (200 μg mL^−1^) in the presence of H_2_O_2_. The data are presented as the means ± SD (*n* = 3).

PDA is endowed with abundant functional groups, which can interact with a variety of molecules or ions through covalent or non‐covalent interactions, and is considered to be an excellent vehicle for drug delivery.^[^ ^20^ ^]^ As shown in **Figure** **1**A, the PM@Mn nanoreactor was constructed by gradually integrating Met and MnO_2_ on the PDA nanoparticles. Initially, referring to previous studies, dopamine monomers were spontaneously polymerized into PDA under alkaline conditions^[^ ^21^ ^]^ and was loaded with Met (PM) through electrostatic and hydrogen bond interactions. Next, the mass ratio of PDA and Met was screened to optimize drug loading. When the mass ratio was 1:2, severe coagulation of the particles was observed, which may be attributed to the disruption of the charge balance in the system by the excess Met (Figure S1A, Supporting Information). The Met loading at different feed ratios was quantified by the ultraviolet subtraction method, and the optimal mass ratio screened out was 1:1, corresponding to a Met loading rate of about 15% (Figure S1D, Supporting Information). Finally, the reduction of phenolic hydroxyl groups was utilized to in situ reduce KMnO_4_ to MnO_2_ to prepare PM@Mn. TEM images revealed that both PDA and PM@Mn were spherical particles with uniform size, and PM@Mn was endowed with better dispersity compared to PDA (Figure 1B). Dynamic light scattering (DLS) results showed that the particle size of PM@Mn did not change significantly compared with PDA and PM, its hydrated particle size was 150.4 nm, which was consistent with the TEM (Figure 1C,D). As shown in Figure 1E, compared with pure PDA and Met‐loaded PM, the zeta potential of PM@Mn gradually increased by about 10 mV. The UV‐Vis absorption spectrum displayed that P@Mn and PM@Mn had absorption bands at 300≈400 nm compared with PDA, which was the same as the surface plasmon band of MnO_2_ reported in the literature,^[^ ^22^ ^]^ and PM@Mn exhibited a characteristic absorption peak at 231 nm, which was attributed to the loading of Met (Figure 1F).^[^ ^23^ ^]^ Moreover, ICP‐OES revealed that the Mn content in PM@Mn was 4.598%. Undoubtedly, good stability and hemocompatibility are the prerequisites to ensure that the nanoparticles reach the target site smoothly through the circulation in vivo. The hydrated particle size of PDA (Figure S2, Supporting Information) and PM@Mn (Figure 1G) in deionized water, 5% FBS, 10% FBS, and RPMI 1640 complete medium had no significant change within 72 h, indicating that PDA and PM@Mn had good stability under simulated physiological conditions. The hemolysis rates of PDA and PM@Mn were both below 2%, illustrating that they had good blood compatibility for intravenous injection (Figure 1H, S3, Supporting Information). Overall, the above results indicated the successful construction of PM@Mn with uniform size, good dispersion, stability, and hemocompatibility.

In order to investigate the photothermal performance of PM@Mn in vitro, a thermal imager was utilized to monitor the temperature variation of the nanoreactor under 808 nm laser irradiation (Figure 1I). The results revealed that the temperature‐raising abilities of PDA and PM@Mn were similar, proving that the introduction of Met and MnO_2_ did not affect the photothermal performance of PDA (Figure S4, Supporting Information). Furthermore, the peak temperature of PM@Mn was positively correlated with the material concentration and irradiation power (Figure 1J,K). Specifically, when the power was 1 W cm^−2^, PM@Mn (200 μg mL^−1^) increased by 53 °C after being irradiated for 5 min. The results of photothermal heating‐cooling cycles showed that the peak temperature of PM@Mn did not change significantly during 5 cycles, exhibiting good photothermal stability (Figure 1L). Subsequently, the ability of PM@Mn for H_2_O_2_ decomposition to O_2_ in vitro was explored by JPBJ‐608 portable dissolved oxygen meter. After adding H_2_O_2_, the oxygen content in PM@Mn gradually increased, while the oxygen content in pure water and PDA did not change significantly, which verified the successful introduction of MnO_2_ in PM@Mn (Figure 1M). Collectively, PM@Mn possesses the desired photothermal conversion and oxygen production properties, promising to inhibit the hypoxia‐adenosinergic axis for enhanced photothermal immunotherapy.

### 2.2 Suppression of Hypoxia‐Adenosinergic Axis in Vitro

**Figure 2:**
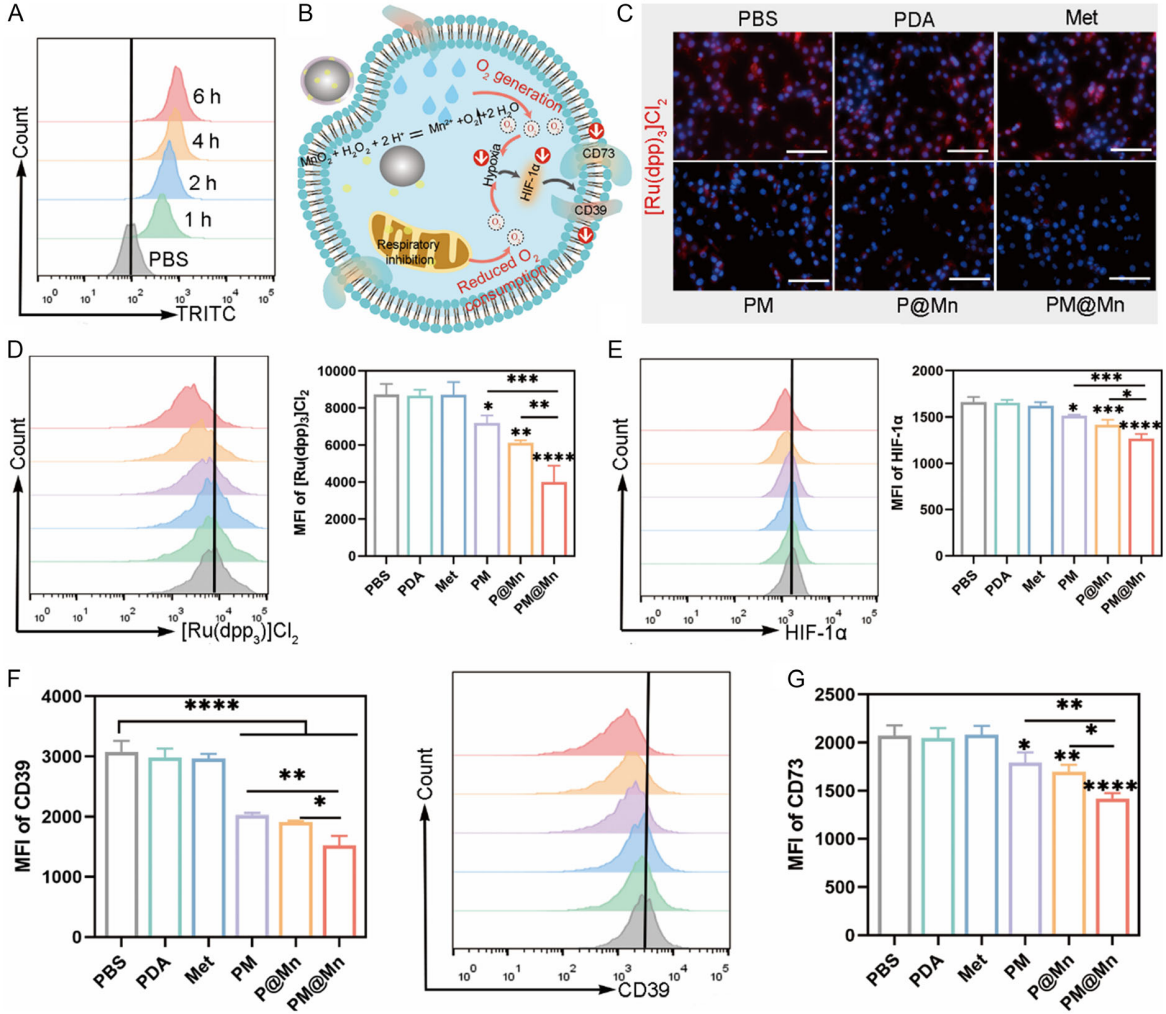
The regulation of PM@Mn on hypoxia‐adenosinergic axis in vitro. A) Representative flow cytometry histogram of TRITC‐PM@Mn and 4T1 after different incubation times (1, 2, 4 and 6 h). B) Schematic diagram of PM@Mn regulating the hypoxia‐adenosinergic axis based on “O_2_ generation with reduced O_2_ consumption motif ” of hypoxia relief. C) Images of PM@Mn and its control groups after incubation with 4T1 taken by living cell workstation (blue: nucleus, red: [Ru(dpp_3_)]Cl_2_, scale bar: 100 μm). Representative flow cytometry histogram and MFI quantitative analysis of D) [Ru(dpp_3_)]Cl_2_ fluorescence, E) PE‐HIF‐1α and F) PE‐CD39 in PM@Mn and its control groups (PBS, PDA, Met, PM and P@Mn, 200 μg mL^−1^ calculated by PDA) after incubation with 4T1. G) MFI quantitative analysis of APC‐CD73 in 4T1 of different treatment groups. The data are presented as the means ± SD (*n* = 3). (* *p* < 0.05, ** *p* < 0.01, *** *p* < 0.001, and **** *p* < 0.0001).

Initially, the cytotoxicity of PM@Mn at different concentrations was measured via CCK‐8. When the concentration of PM@Mn was in the range of 400 μg mL^−1^, the cell viability of 293 T was basically not affected (Figure S5A, Supporting Information). Nevertheless, when the concentration of PM@Mn was higher than 300 μg mL^−1^, the cell viability of 4T1 was slightly lower than 80% (Figure S5B, Supporting Information), which may be related to the inhibition of mitochondrial respiration by Met on hyperproliferative tumor cells.^[^ ^24^ ^]^ Overall, PM@Mn was almost non‐cytotoxic without laser irradiation. Next, to explore the cellular uptake of nanoreactor, flow cytometry was employed to quantify TRITC‐labeled PM@Mn incubated with 4T1 for different times. The flow cytometry results revealed that PM@Mn could be efficiently internalized as the incubation time increased within 6 h (**Figure** **2**A). As shown in Figure S6B (Supporting Information), when incubated with TRITC‐PM@Mn for 4 h or 6 h, there was no significant difference in the mean fluorescence intensity (MFI). Hence, laser irradiation was performed after 4 h of incubation in subsequent experiments.

Since the fluorescence of [Ru(dpp_3_)]Cl_2_ is significantly quenched when encountering oxygen molecules, it is utilized to characterize cellular hypoxia.^[^ ^25^ ^]^ Before exploring the hypoxia relief of PM@Mn on 4T1, the effect of different concentrations of free Met was verified using the hypoxia probe [Ru(dpp_3_)]Cl_2_. The MFI of the low concentration treatment group (100 μg mL^−1^) was not significantly different from that of the PBS group (Figure S8, Supporting Information). Only when the concentration of Met was higher than or equal to 200 μg mL^−1^, the corresponding MFI decreased significantly, but it had no significant difference from that of the high concentration treatment group (500 μg mL^−1^). The results suggested that free Met could relieve hypoxia only at a higher concentration, and mitochondrial respiration suppression alone has a limited effect on hypoxia remission. Subsequently, the hypoxia relief of PM@Mn and its control groups (PBS, PDA, Met, PM, and P@Mn) on 4T1 was evaluated by fluorescence colocalization and flow cytometry. The fluorescence intensity of PM group, P@Mn group and PM@Mn group decreased to varying degrees. Among them, the red fluorescence of PM@Mn group was the weakest, indicating that PM@Mn had the most remarkable effect on improving hypoxia (Figure 2C,D). Next, we evaluated the duration of oxygen production by PM@Mn at the cellular level by flow cytometry. The fluorescence intensity of [Ru(dpp_3_)]Cl_2_ decreased significantly after being co‐cultured with PM@Mn and 4T1 for 2 h. Moreover, with the extension of incubation time, there was no significant difference in fluorescence intensity at 8, 16 and 24 h (Figure S9, Supporting Information). The results indicate that PM@Mn, combines catalytic oxygen production with reduced endogenous oxygen consumption, takes account of oxygen level and oxygen persistence simultaneously, thus exhibiting excellent hypoxia relief ability. The stable expression of HIF‐1α can indirectly reflect the degree of intracellular hypoxia, and HIF‐1α is a crucial marker of hypoxia‐activated related pathways.^[^ ^26^ ^]^ The quantitative results of flow cytometry revealed that the experimental group PM@Mn was more effective in down‐regulating the expression of HIF‐1α compared with the PM group and the P@Mn group, which was consistent with the overall trend of the hypoxia probe experiment (Figure 2E). Western Blot results also revealed that the expression level of HIF‐1α in the PM@Mn group was significantly decreased compared with the untreated control group (Figure S10, Supporting Information). Generally, compared with only mitochondrial respiration suppression or H_2_O_2_ catalytic decomposition into O_2_, PM@Mn can relieve tumor cell hypoxia more significantly, which also proves the superiority of catalytic oxygen production with reduced endogenous oxygen consumption on hypoxia relief.

Ectonucleotidases CD39 and CD73 are pivotal enzymes in the process of ATP metabolism to adenosine, and the expression of HIF‐1α affects the levels of CD39 and CD73.[^6^, ^13^] The expression of CD39 and CD73 in 4T1 of different treatment groups was evaluated by flow cytometry. Compared with the PBS group, the levels of CD39 and CD73 in the PM group, P@Mn group and PM@Mn group were down‐regulated to varying degrees. Among them, the expression of CD39 and CD73 in the PM@Mn group was the lowest, which reduced by 50.7% and 31.5% compared with the PBS control group, respectively (Figure 2F,G, and S11, Supporting Information). These results illustrated that PM@Mn could significantly suppress the hypoxia‐adenosinergic axis in vitro based on above mentioned motif of hypoxia relief.

### 2.3 Evaluation of PM@Mn‐Mediated Photothermal ICD

**Figure 3:**
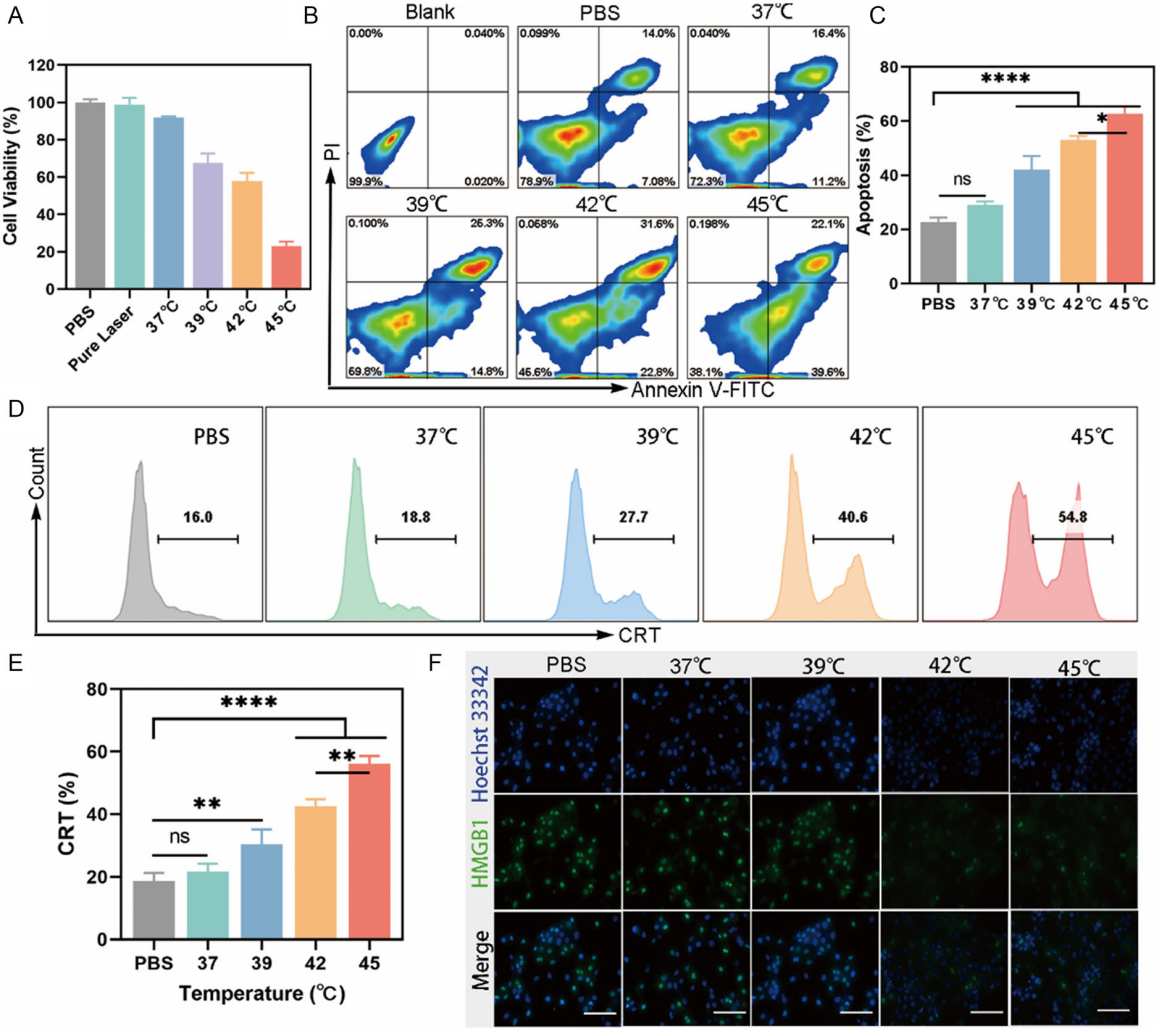
Evaluation of PM@Mn‐mediated photothermal ICD in 4T1. A) The cell viability of 4T1 under different irradiation temperatures by CCK‐8 (*n* = 5). B) Representative flow cytometry plots of apoptosis and necrosis of 4T1 and C) the analysis of apoptosis rate under different irradiation temperatures (37, 39, 42 and 45 °C). D) Representative flow cytometry histogram and E) the percentage of expressing CRT in 4T1 cells under different irradiation temperatures (37, 39, 42 and 45 °C). F) The pictures of HMGB1 expression in 4T1 under different irradiation temperatures observed by the living cell workstation (scale bar: 100 μm). The data are presented as the means ± SD (*n* = 3). (ns, not significant, * *p* < 0.05, ** *p* < 0.01 and **** *p* < 0.0001).

CCK‐8 and apoptosis assay kit were utilized to further investigate the photothermal killing effect of PM@Mn on 4T1 cells. As shown in **Figure** **3**A, the cell viability of 4T1 in the Pure Laser group was not significantly different from that in PBS group. Nevertheless, the cell viability of 4T1 decreased gradually with the increase of irradiation temperature. When the temperature was 45 °C, the corresponding cell viability was only 23%, indicating that the killing effect of PM@Mn on 4T1 mainly depended on the photothermal conversion. Apoptosis assay displayed that the apoptosis rate of 4T1 cells rose with increasing irradiation temperature, and the apoptosis rate at 39, 42 and 45 °C was 1.86‐, 2.34‐, and 2.77‐fold versus the PBS group, respectively, proving that 42≈45 °C could induce significant apoptosis and necrosis of tumor cells (Figure 3B,C).

Induction of ICD is crucial for PTT to activate adaptive immune response based on local ablation of tumor.^[^ ^2^ ^]^ During the ICD process, calreticulin (CRT) will be translocated and exposed to the surface of the cell membrane, releasing the “eat me” signal to immune cells, and accompanied by the leakage of high mobility group protein B1 (HMGB1) from the nucleus to the extracellular.^[^ ^27^ ^]^ Therefore, whether the thermal effect of PM@Mn can induce ICD of 4T1 cells was further explored via detecting the expression of ICD markers (Figure 3D,E). With the increase of irradiation temperature, the green fluorescence signal of CRT on the cell membrane of 4T1 heightened (Figure S12, Supporting Information). When irradiated at 45 °C, the percentage of 4T1 cells exposed to CRT on the cell membrane was about 3.01‐fold that of the PBS group through flow cytometry analysis. Moreover, as the irradiation temperature increased, the overlap of the HMGB1 green fluorescence signal with the nucleus (blue) decreased, indicating that 42≈45 °C could promote the leakage of HMGB1 from the nucleus (Figure 3F). The above results demonstrated that PM@Mn could induce ICD of tumor cells through photothermal conversion when irradiated by 808 nm laser, and the degree of ICD increased with the rise of irradiation temperature in a certain range. In order to maximize the induction of ICD and immune activation, and to avoid damage to surrounding tissues caused by high heat, 45 °C was selected for subsequent related experiments.

### 2.4 Investigation of PM@Mn on Remodeling Adenosinergic Axis for Enhanced Photothermal Immunotherapy in Vitro

**Figure 4:**
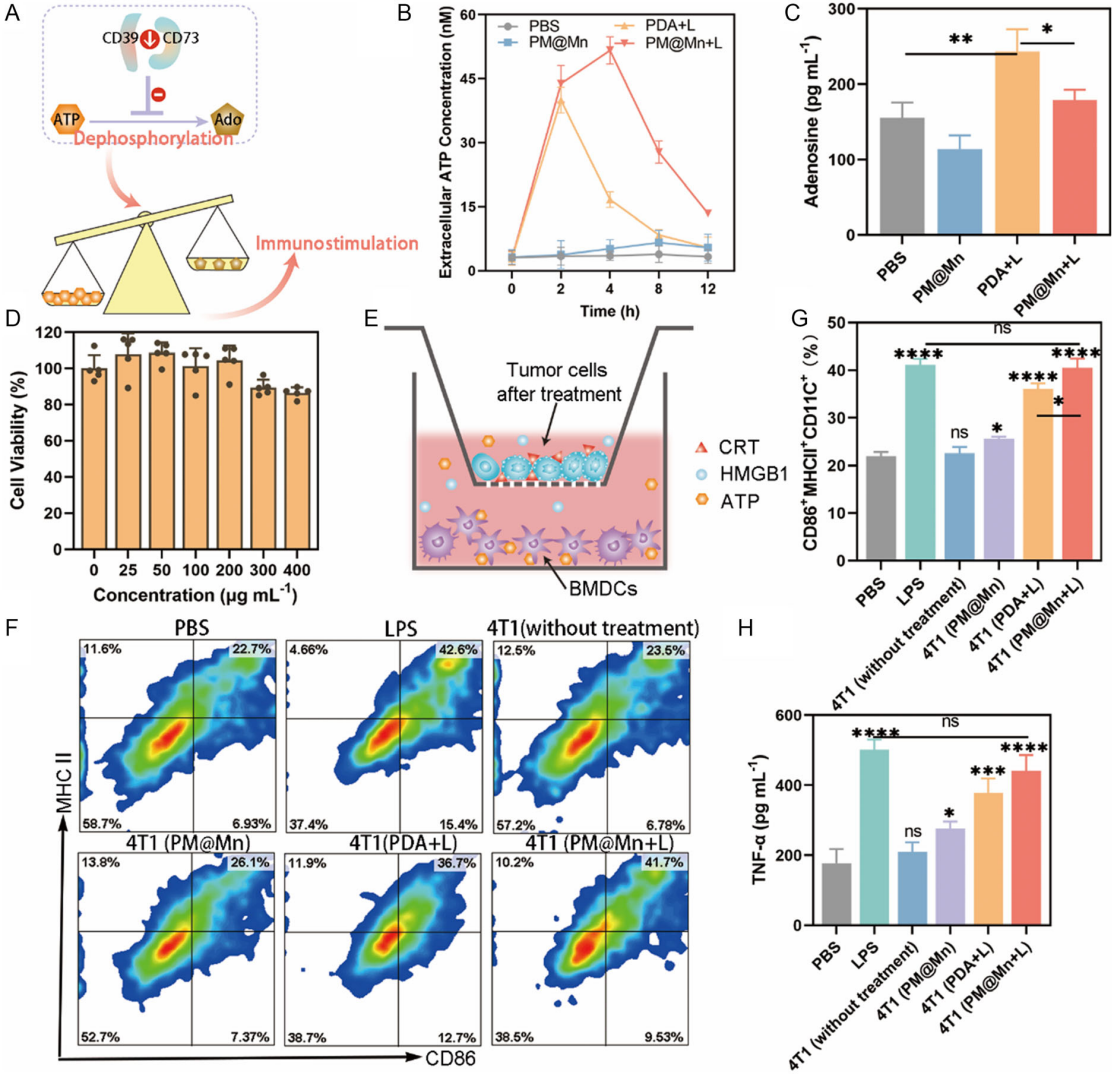
Investigation of PM@Mn for enhanced photothermal immunotherapy in vitro. A) Schematic diagram of modulating ATP‐based adenosinergic axis. Contents of extracellular B) ATP and C) adenosine in 4T1 under different treatments (PBS, PM@Mn, PDA + L and PM@Mn + L, 200 μg mL^−1^ calculated by PDA; “L” means irradiation of 808 nm laser at 45 °C for 8 min). D) Cell viability after different concentrations of PM@Mn incubated with 4T1 for 24 h (*n* = 5). E) Schematic diagram of BMDCs maturation induced by Transwell co‐incubation experiment. F) Representative flow cytometry plots and G) the percentages of CD86^+^MHC II^+^ expression in BMDCs under different treatments (PDA and PM@Mn, 200 μg mL^−1^ calculated by PDA; 1 μg mL^−1^ LPS; “L” means irradiation of 808 nm laser at 45 °C for 8 min). H) Content of TNF‐α in BMDCs’ supernatant under different treatments. The data are presented as the means ± SD (*n* = 3). (ns, not significant, * *p* < 0.05, ** *p* < 0.01, *** *p* < 0.001, and **** *p* < 0.0001).

When tumor cells undergo apoptosis, ATP is released from intracellular to extracellular, which facilitates the recruitment of DCs to the site of apoptosis, and promotes their maturation and activation, thereby stimulating specific antitumor immunity.^[^ ^28^ ^]^ Nevertheless, the adenosinergic axis in tumors rapidly metabolizes the immune‐activating ATP to the immunosuppressive adenosine, thereby weakening the ICD‐triggered immune response.[^4^, ^29^] Assay kits were employed to quantitatively analyze the contents of ATP and adenosine after PDA or PM@Mn treatment with or without laser irradiation to explore whether PM@Mn under laser irradiation could remodel adenosinergic axis. As shown in **Figure** **4**B, the extracellular ATP of 4T1 after laser irradiation increased in a short period, but was quickly metabolized. Compared with the PDA + L group (“L” refers to 808 nm laser irradiation at 45 °C for 8 min), the extracellular ATP metabolism in the PM@Mn + L group was relatively slow. After 12 h of treatment, the ATP in the supernatant of 4T1 cells in the PM@Mn + L group was about 4‐fold that of the PBS group. The results of adenosine detection revealed that after 12 h of treatment, the adenosine content in the PDA + L group increased, which was 1.57‐fold that of the PBS group, but the adenosine content in the PM@Mn + L group decreased versus the PDA + L group (Figure 4C). Based on the preclinical biochemical‐immunological studies^[^ ^11^ ^]^ and our results in Part 3.2, we speculated that the decrease of adenosine content in the PM@Mn + L group versus the PDA + L group was mainly attributed to the downregulation of CD39 and CD73 by PM@Mn‐mediated hypoxic relief, thereby suppressing adenosine production. The above results indicated that PM@Mn could increase the extracellular ATP level after laser irradiation, and could inhibit the metabolism of ATP into adenosine, which is expected to enhance the immune response via remodeling the adenosinergic axis.

As professional antigen‐presenting cells, the maturation and activation of DCs are critical for initiating adaptive antitumor immunity.^[^ ^30^ ^]^ Firstly, the effects of different concentrations of PM@Mn on the cell viability of BMDCs were evaluated by CCK‐8. When the concentration of PM@Mn was in the range of 400 μg mL^−1^, the cell viability of BMDCs was above 85%, indicating that PM@Mn has good biocompatibility (Figure 4D). Subsequently, the Transwell co‐incubation system was designed to explore the effect of PTT‐induced ICD on the maturation of DCs, and whether PM@Mn could enhance the immune response via remodeling adenosinergic axis in vitro. Concretely, differently treated 4T1 were inoculated on the upper layer of the Transwell system, BMDCs were inoculated on the lower layer (Figure 4E), and untreated and LPS‐treated (1 μg mL^−1^) BMDCs were utilized as negative and positive controls, respectively. CD86 and MHCII are well known as specific biomarkers to characterize the maturation of BMDCs. The results of flow cytometry displayed that the percentage of CD86^+^MHC II^+^ BMDCs in PM@Mn + L group was 1.85‐, 1.58‐ and 1.12‐fold of those in the PBS group, PM@Mn group and PDA + L group, respectively, and was not significantly different from that in the LPS positive control group (Figure 4F,G). This demonstrated that photothermal‐triggered ICD could promote the maturation of BMDCs, and PM@Mn treatment improved the maturation of BMDCs induced by PTT alone. Next, the level of inflammatory cytokine TNF‐α, an indicator of DCs activation, in the supernatants of different treatment groups was detected by Elisa. As shown in Figure 4H, the content of TNF‐α in the supernatant of the PM@Mn + L group was higher than that of the PDA + L group, and there was no significant difference compared with the LPS positive control group. The overall trend of Elisa was consistent with the results of flow cytometry experiments. Together, PM@Mn can enhance photothermal ICD‐induced antitumor immunity via inhibiting the hypoxia‐adenosinergic axis in vitro.

### 2.5 Evaluation of 4T1 Tumor Growth Suppression in Vivo

**Figure 5:**
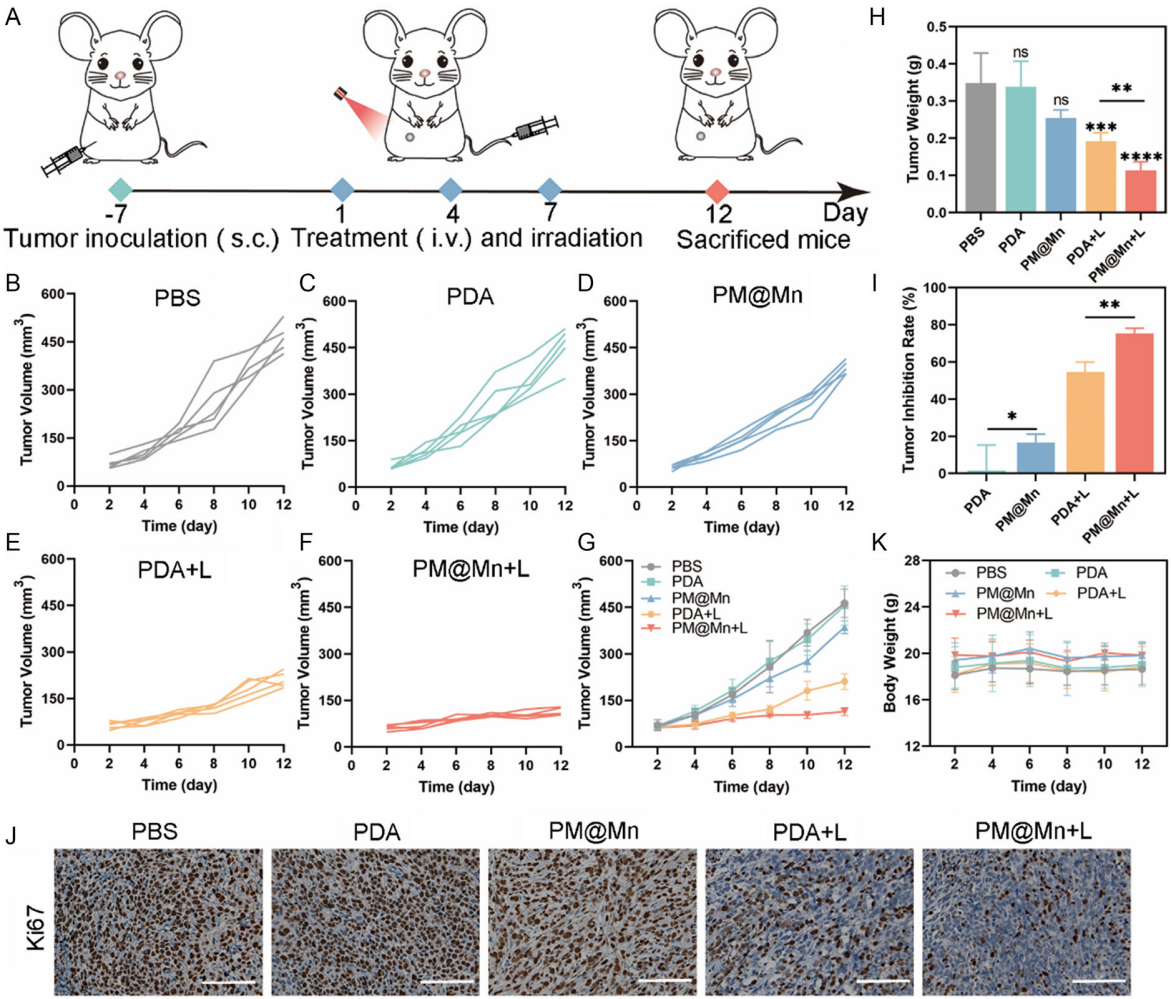
Inhibition of tumor growth in different treatment groups. A) Schematic diagram of 4T1 tumor‐bearing mouse model establishment and treatment. B–G) The changes of tumor volume in different treatment groups. H) Tumor weight in different treatment groups after monitoring end. I) Tumor inhibition rate in other treatment groups compared with the PBS group. J) Expression of Ki67 in different treatment groups (scale bar: 100 μm). K) Weight changes of mice in different groups during treatment. The data are presented as the means ± SD (*n* = 5). (ns, not significant, * *p* < 0.05, ** *p* < 0.01, *** *p* < 0.001, and **** *p* < 0.0001).

The hypoxia relief level of PM@Mn is the basis of its ability to suppress the hypoxia‐adenosinergic axis and thus exert anti‐tumor effects. Therefore, we first assessed the duration of oxygen production by PM@Mn in tumor tissues. As shown in Figure S15 (Supporting Information), The changing trend of fluorescence intensity of [Ru(dpp_3_)]Cl_2_ after injection PM@Mn for different times was consistent well with the results at cellular level (Figure S9, Supporting Information). Together, these results indicate that PM@Mn can induce efficiently oxygenation after injection PM@Mn for 8 h, and the oxygen level tends to be stable thereafter until 24 h. Next, we continued to investigate the antitumor effect of PM@Mn in vivo. Specifically, the antitumor effect of PM@Mn under 808 nm laser irradiation in vivo was evaluated in 4T1 orthotopic tumor‐bearing mouse model. Tumor‐bearing mice were randomly divided into 5 groups (PBS, PDA, PM@Mn, PDA + L, and PM@Mn + L). As shown in **Figure** **5**A, 200 μL of PDA or PM@Mn (dose of 20 mg kg^−1^ calculated by PDA) were intravenously injected on the 1st, 4th, and 7th day, and 808 nm laser was utilized to irradiate the PDA + L group and PM@Mn + L group 2 h after administration (45 °C, 8 min). The volume and weight of tumors in the PDA group had no significant differences from those in the PBS group (Figure 5G,H), while the tumor growth in the PM@Mn group, PDA + L group, and PM@Mn + L group was inhibited to varying degrees (Figure 5D–F). Among them, the PM@M + L group exhibited the most conspicuous therapeutic effect, and the inhibition rate reached 75.4% (Figure 5I). Strikingly, tumor growth was accelerated in the PDA + L group after cessation of treatment, while tumor growth suppression was maintained in the PM@Mn + L group (Figure 5G), which may be attributed to the improvement of immunosuppressive microenvironment induced by hypoxia‐adenosinergic axis modulation. Moreover, the tumor tissue of PM@Mn + L group showed the lowest expression of Ki67 (Figure 5J), which was closely related to tumor proliferation.^[^ ^31^ ^]^ Conclusively, PTT can ablate tumors, and remodeling hypoxia‐adenosinergic axis can cooperate with PTT to exert an obvious antitumor effect.

Notably, biosafety during treatment is critical to assessing the application potential of a treatment regimen. As shown in Figure 5K, the body weight of mice in each group did not change significantly during the treatment. Besides, H&E staining results displayed that heart, liver, spleen, lung, and kidney of mice in each group had no obvious organ damage compared with the PBS group (Figure S16, Supporting Information). The corresponding liver and kidney function indexes, the contents of alanine aminotransferase (ALT), aspartate aminotransferase (AST), and creatinine (CRE), were all within the normal range (Figure S17, Supporting Information). These results indicated that PDA and PM@Mn had good biosafety during treatment.

### 2.6 Evaluation of Suppressing Effect on Hypoxia‐Adenosinergic Axis in Vivo

**Figure 6:**
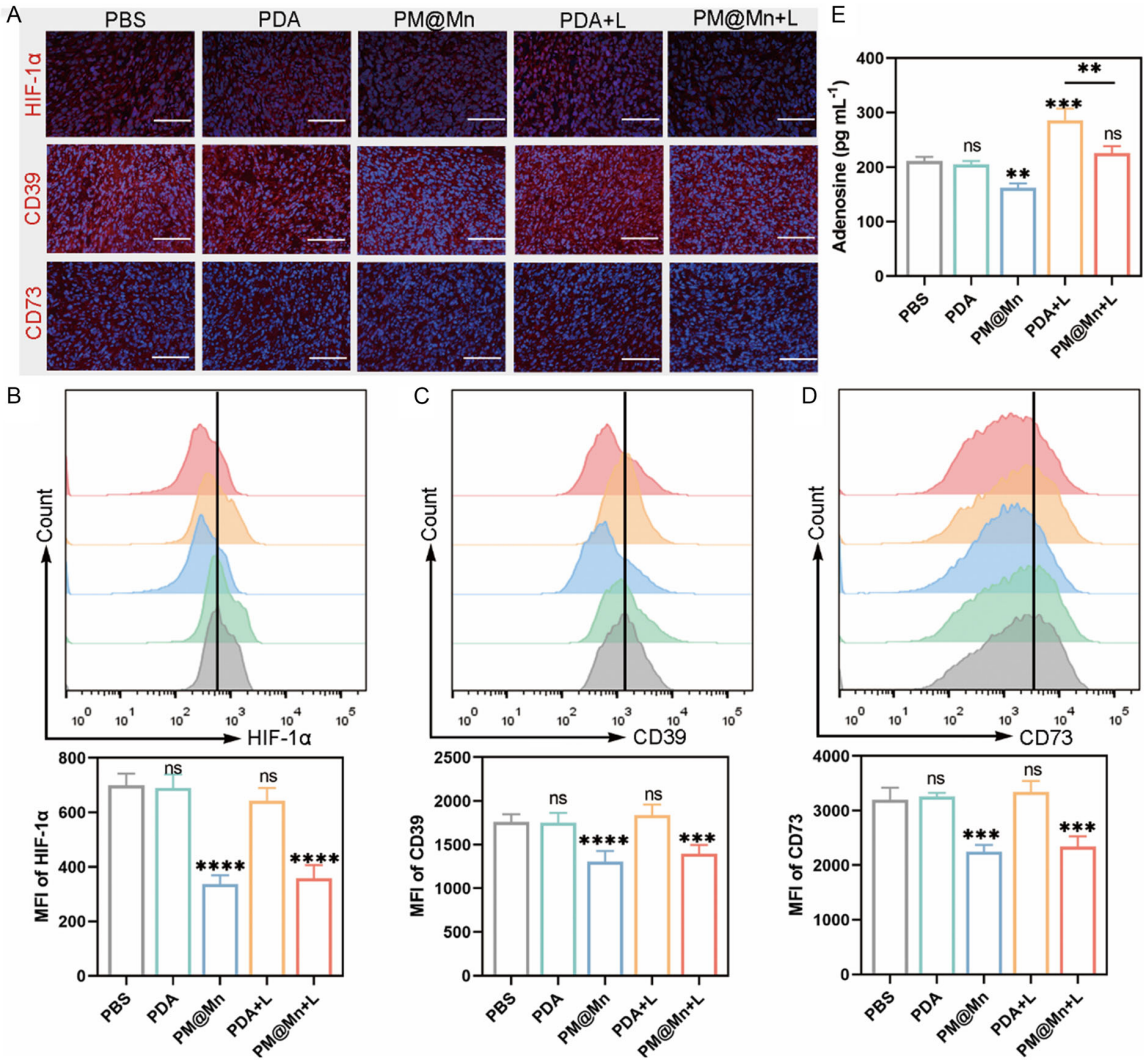
Regulation performance of PM@Mn on hypoxia‐adenosinergic axis in tumor tissues. A) Immunofluorescence co‐localization of HIF‐1α, CD39 and CD73 in tumor tissues of different treatment groups (scale bar: 100 μm). Representative flow cytometry histogram and corresponding MFI of B) HIF‐1α, C) CD39 and D) CD73 in different treatment groups (PBS, PDA, PM@Mn, PDA + L and PM@Mn + L). E) The content of adenosine in tumor tissues of different treatment groups. The data are presented as the means ± SD (*n* = 5). (ns, not significant, ** *p* < 0.01, *** *p* < 0.001, and **** *p* < 0.0001).

CD39 and CD73 are critical phosphatases in the process of ATP metabolism to adenosine, and are widely distributed in tumor tissues.^[^ ^29^ ^]^ Hypoxic microenvironment can activate adenosinergic axis through HIF‐1α‐dependent upregulation of CD39 and CD73 expression.^[^ ^13^, ^32^ ^]^ Given this, the effect of PM@Mn on the hypoxia‐adenosinergic axis in vivo was explored. Firstly, the expression levels of adenosine metabolism pivotal indicators in tumor tissues of each group were investigated via immunofluorescence. The nuclei was marked in blue, and HIF‐1α, CD39 and CD73 were marked in red, respectively. As shown in **Figure** **6**A, the red fluorescence of HIF‐1α, CD39, and CD73 in the PDA group and PDA + L group were not significantly different from those in the PBS group, while the corresponding red fluorescence of the PM@Mn group and PM@Mn + L group weakened, preliminarily indicating that PM@Mn could down‐regulate the expression of adenosine metabolism pivotal indicators. Similarly, flow cytometry results displayed that the MFI of HIF‐1α, CD39 and CD73 in the PDA group and PDA + L group had no significant difference compared with the PBS group (Figure 6B–D). Nevertheless, the corresponding MFI of PM@Mn group and PM@Mn + L group decreased significantly, which was consistent with the results of immunofluorescence assay, proving that PM@Mn can suppress the hypoxia‐adenosinergic axis in vivo.

Subsequently, the tumor tissue was ground in PBS containing an adenosine degrading enzyme inhibitor (weight to volume ratio: 1:5), the supernatant was collected by centrifugation, and the content of adenosine in the tumor tissue of each group was determined by Elisa. As shown in Figure 6E, the content of adenosine in the PDA + L group was 1.35‐fold that of the PBS group, while the content of adenosine in the PM@Mn + L group was significantly lower than that in the PDA + L group (*p* < 0.01). These results suggested that photothermal treatment increased the accumulation of adenosine in tumor tissues, and suppression of the hypoxia‐adenosinergic axis could attenuate the upregulation of adenosine induced by photothermal treatment.

### 2.7 Analysis of Antitumor Immune Mechanism in Vivo

**Figure 7:**
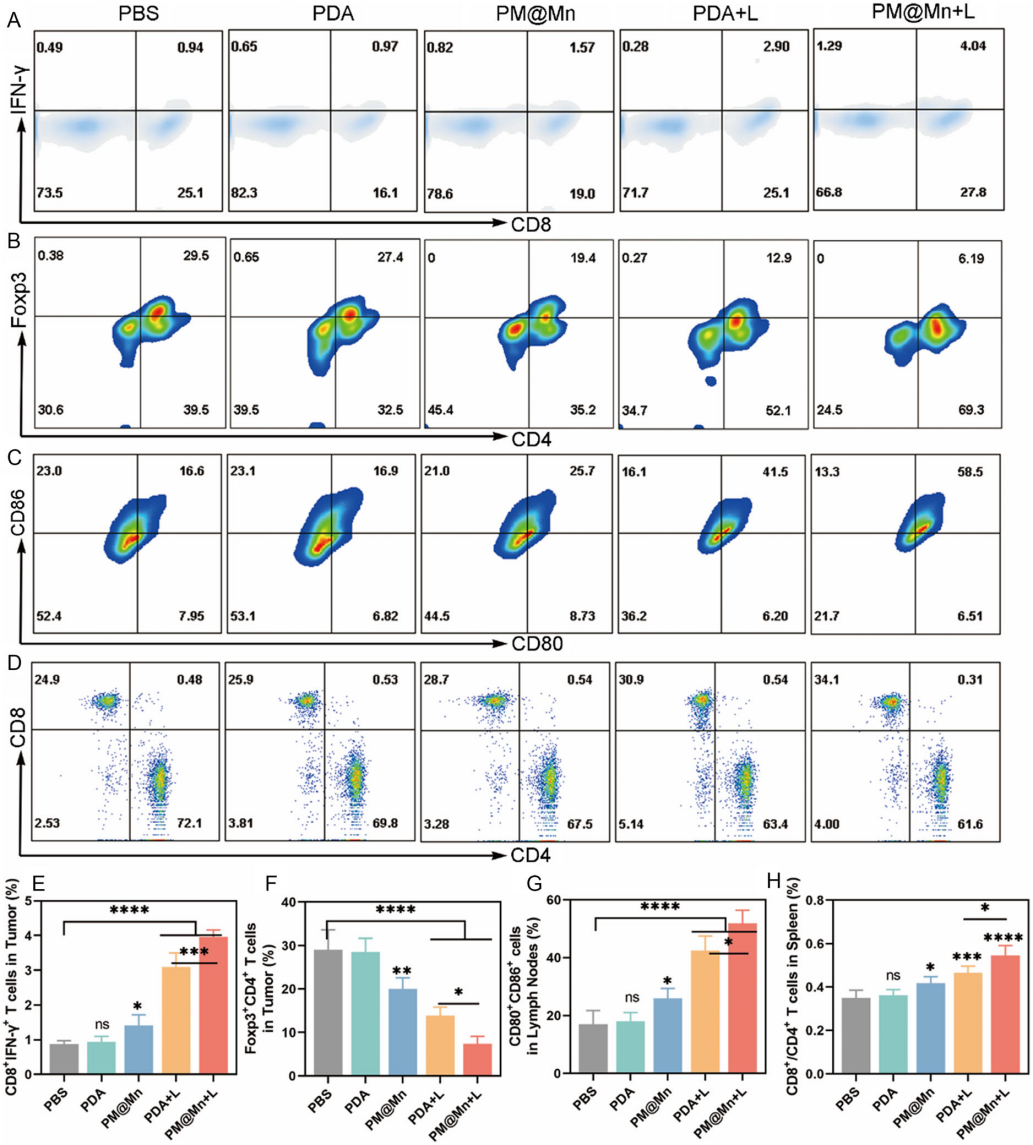
Regulation of antitumor immunity by PM@Mn under laser irradiation. A) Representative flow cytometry plots of CD8^+^IFN‐γ^+^ T cells and B) Foxp3^+^CD4^+^ T cells (gated on CD3^+^ T cells) infiltration in tumors of different treatment groups. C) Representative flow cytometry plots of CD80^+^CD86^+^ DCs (gated on CD11c^+^ cells) in lymph nodes of different treatment groups. D) Representative flow cytometry plots of the proportion of CD8^+^ T cells and CD4^+^ T cells (gated on CD3^+^ T cells) in spleen of different treatment groups. E) The percentages of CD8^+^IFN‐γ^+^ T cells and F) Foxp3^+^CD4^+^ T cells infiltration in tumors. G) The percentages of the infiltration of CD80^+^CD86^+^ DCs in lymph nodes. H) The analysis of CD8^+^/CD4^+^ T cells ratio in spleen of different treatment groups. The data are presented as the means ± SD (*n* = 5). (ns, not significant, * *p* < 0.05, *** *p* < 0.001, and **** *p* < 0.0001).

The effect of PM@Mn under laser irradiation on tumor growth suppression and adenosine metabolism prompted us to continue to explore the underlying immune regulatory mechanism. CD8^+^ T cells are the main force of adaptive antitumor immunity.^[^ ^33^ ^]^ Thus, the infiltration of T lymphocytes in tumor tissues of different groups was analyzed by flow cytometry. The PM@Mn + L group induced the highest ratio of CD8^+^/CD4^+^ T cells, which was 3.19‐fold, 1.91‐fold, and 1.27‐fold that of the PBS group, PM@Mn group, and PDA + L group, respectively (Figure S18, Supporting Information). Activated CD8^+^ T cells mainly secrete IFN‐γ to exert antitumor activity.^[^ ^34^ ^]^ Similarly, the proportion of CD8^+^IFN‐γ^+^ T cells in the PM@Mn + L group also reached a peak, which was 4.51‐fold that of the PBS group (**Figure** **7**A,E). The striking infiltration and activation of cytotoxic T lymphocytes in the PM@Mn + L group may be attributed to the induction of photothermal ICD and the relief of adenosine immunosuppression. The number of immunosuppressive cells in tumor tissue is also closely related to the level of immune activation. Compared with the PBS group, the proportion of Foxp3^+^CD4^+^ T cells (Tregs) in the PM@Mn + L group was remarkably decreased (Figure 7B,F). These results demonstrate that PM@Mn can effectively modulate the infiltration of T lymphocytes in tumor tissues and enhance photothermal immunotherapy.

Additionally, the effect of PM@Mn under 808 nm laser irradiation on systemic antitumor immunity was analyzed. As professional antigen‐presenting cells, the maturation of DCs is critical for the presentation of tumor‐associated antigens and the initiation of adaptive antitumor immunity.^[^ ^30^ ^]^ As shown in Figure 7C,G and S19 (Supporting Information), the expression of DCs maturation markers (CD80^+^CD86^+^ and CD86^+^MHC II^+^) in the lymph nodes of the PM@Mn + L group was the highest, which was 3.06‐fold and 1.87‐fold that of the PBS group, respectively. This indicated that PM@Mn synergistically with PTT could dramatically stimulate the maturation of DCs in lymph nodes. As the major immune organ in the body, the spleen is rich in immune cells. Thus, T lymphocyte phenotypes and MDSCs in the spleen were also examined. Compared with the PBS group, PM@Mn + L increased the ratio of CD8^+^ /CD4^+^ T cells from 0.35 to 0.55 (Figure 7D,H), while the percentage of CD11b and Gr‐1 double‐positive cells (MDSCs) was significantly decreased (*p* < 0.0001) (Figure S20, Supporting Information), indicating that PM@Mn synergistically with PTT effectively activates adaptive antitumor immunity.

Taken together, PM@Mn synergistically with PTT can not only trigger remarkable local immunity in tumors, but also effectively activate systemic antitumor immunity in lymph nodes and spleen.

## 3 Conclusion

Summarily, we developed a strategy to alleviate the immunosuppressive microenvironment and thus boost photothermal immunotherapy via inhibiting the hypoxia‐adenosinergic axis. Based on the photothermal properties, carrier function and reducibility of PDA, PM@Mn nanoreactor with uniform size, good dispersity, and stability was successfully constructed. In vitro and in vivo, PM@Mn induced photothermal ICD, effectively down‐regulated the expression of adenosine metabolism pivotal indicators HIF‐1α, CD39, and CD73 through the “increase O_2_ generation and decrease O_2_ consumption” motif of hypoxia relief, thereby reducing the accumulation of the immunosuppressive metabolite adenosine. Notably, PM@Mn under laser irradiation increased the proportion of CD8^+^IFN‐γ^+^ T cells in tumors to 4.51‐fold that of the PBS group, while strikingly down‐regulated the proportion of immunosuppressive Tregs (Foxp3^+^CD4^+^ T cells), showing a tumor inhibition rate of 75.4%. Similarly, this metabolic immunotherapy resulted in the activation of systemic antitumor immunity in the lymph nodes and spleen. Suppressing hypoxia‐adenosinergic axis can also be extended to other therapeutic modalities for inducing ICD. As a proof of concept, this project enriches the research on the modulation of adenosine metabolism, and provides a new avenue for regulating tumor metabolism to enhance ICD‐triggered antitumor immunity.

## 4 Experimental Section

4.1

4.1.1

##### Synthesis of PM@Mn Nanoreactor

Under alkaline conditions, dopamine can spontaneously polymerize to form PDA nanoparticles with oxidants such as oxygen, and the detailed preparation scheme referred to previous research.^[^ ^21^ ^]^ Under mild magnetic stirring conditions, the Met solution (2.5 mg mL^−1^) was added dropwise to the PDA solution (1 mg mL^−1^), so that the mass ratio of PDA to Met was 1:1. After continuous stirring for 6 h, the free Met was removed by centrifugation and washing with deionized water, and Met‐loaded PDA nanoparticles (PM) were obtained. The construction of the PM@Mn nanoreactor took advantage of the reducibility of PDA, and the method drew on the previous research.^[^ ^35^ ^]^ Specifically, under mild magnetic stirring conditions, the KMnO_4_ solution (0.5 mg mL^−1^) was slowly added dropwise to the PDA or PM solution (1 mg mL^−1^), so that the mass ratio of PDA to KMnO_4_ was 5:1. After the overnight reaction, P@Mn or PM@Mn were obtained by centrifugation and washing with deionized water. Finally, the purified nanoparticles of different components were stored at 4 °C for subsequent use, and the specific concentrations used in the experiments were quantified by PDA.

##### Characterization of PM@Mn Nanoreactor

The hydrated particle size, polydispersity index (PDI) and Zeta potential of PDA, P@Mn, and PM@Mn were measured by dynamic light scattering (Nano‐ZS 90, Malvern). The morphology and size of nanoparticles were observed by transmission electron microscope (TEM, JEM‐1230). The full‐wavelength scanning of the samples to be tested were performed by a UV‐visible spectrophotometer (UV‐Vis, Cry50), and the changes of the absorption peaks of PDA, PM, and PM@Mn were compared. The content of Mn element in PM@Mn was quantitatively analyzed by inductively coupled plasma optical emission spectrometer (ICP‐OES, Agilent 725).

##### Stability Investigation of PDA and PM@Mn

PDA and PM@Mn were diluted to 0.2 mg mL^−1^, and three parallels were set for each sample. At predetermined time points (0, 1, 2, 4, 6, 12, 24, 48, and 72 h), 20 μL samples were taken for appropriate dilution, and dynamic light scattering was used to observe the particle size variation of nanoreactors in water, 5% FBS, 10% FBS, and RPMI‐1640 complete medium.

##### Hemolysis Investigation of PM@Mn

The hemocompatibility of nanoparticles was investigated by quantitatively analyzing the hemolysis of different concentrations of PDA and PM@Mn (25, 50, 100, 200, 300, and 400 μg mL^−1^) incubated with blood by a microplate reader. Specifically, using deionized water as a positive control and PBS as a negative control, samples of different concentrations were mixed with equal volumes of 5% erythrocyte suspension prepared in advance, then incubated at 37 °C for 4 h, and the supernatant was collected by centrifugation. The absorbance at 540 nm of the supernatants of each group was then measured with a microplate reader. The formula for calculating the hemolysis rate was as follows:

(1) $$HemolysisRatio\%=\frac{{\text{OD}}_{\text{sample}}-{\text{OD}}_{\text{negative}}}{{\text{OD}}_{\text{positive}}-{\text{OD}}_{\text{negative}}}\times 100\%$$

##### Photothermal Performance Investigation of PM@Mn

A thermal imager was employed to monitor the temperature variation of PM@Mn under 808 nm laser irradiation to investigate the photothermal properties. Specifically, a laser (0.75 W) was employed to continuously irradiate PDA or PM@Mn (200 μg mL^−1^) for 10 min. Meanwhile, a thermal imager was utilized to monitor and record the temperature variation in real time to investigate the temperature increment with the irradiation time. Moreover, the temperature increment of PM@Mn under different concentrations (25, 50, 100, and 200 μg mL^−1^) and different power (0.25, 0.5, 0.75, and 1 W cm^−2^) were investigated respectively. Finally, the laser was continuously maintained irradiation with a fixed power (0.75 W cm^−2^) for 5 min, and then the laser was turned off to allow the PM@Mn (200 μg mL^−1^) to naturally cool down to the initial temperature. Repeat five heating‐cooling cycles to explore the photothermal cycle performance of PM@Mn.

##### Oxygen Production Performance of PM@Mn in Vitro

The oxygen content was monitored in real time by a portable dissolved oxygen meter (JPBJ‐608) to investigate the catalytic oxygen production performance of PM@Mn in vitro. Specifically, the dissolved oxygen meter was inserted into the solution to be tested (200 μg mL^−1^), and then a certain volume of H_2_O_2_ (30 wt%) was carefully added to keep the concentration of H_2_O_2_ 50 mM, and the displayed value of meter was recorded in real time and monitored continuously for 30 min.

##### Cytotoxicity Evaluation in Vitro

The toxicity of different concentrations of PM@Mn to normal cell 293 T and tumor cell 4T1 was determined via CCK‐8 assay kit. Concretely, cells were seeded in 96‐well plates (1 × 10^4^ cells per well) and cultured overnight. The supernatant was replaced with the fresh complete medium containing different concentrations of PM@Mn (25, 50, 100, 200, 300, or 400 μg mL^−1^). After incubation for 24 h, the cells were washed with PBS, and were added 100 μL 10% CCK‐8 to continue incubation for 1≈2 h. After incubation, the absorbance of each well at 450 nm was detected with a microplate reader, and the cell viability was calculated according to the following formula:

(2) $$CellViability\left(\%\right)=\frac{{\text{OD}}_{\text{sample}}-{\text{OD}}_{\text{blank}}}{{\text{OD}}_{\text{control}}-{\text{OD}}_{\text{blank}}}\times 100\%$$

##### Cellular Uptake Evaluation

Rhodamine B isothiocyanate (TRITC) can undergo amide reaction with amino groups under weak alkaline conditions, be conjugated to PDA, and further be prepared dye‐labeled PM@Mn to explore the cellular uptake. Concretely, 4T1 was seeded in a 24‐well plate (1 × 10^5^ cells per well) and cultured overnight. Subsequently, the medium was substituted with fresh medium containing TRITC‐PM@Mn (200 μg mL^−1^) and incubated for different time (1, 2, 4, or 6 h). After incubation, the cells were washed with PBS and stained with Hoechst 33 342, and then fluorescent colocalization images were taken using a live cell workstation (Lionheart FX, BioTek). For qualitative analysis, the above co‐incubated cells were digested and collected, and then detected by flow cytometry (FACSVerse, BD).

##### Hypoxia Evaluation in Vitro

Oxygen indicating probe [Ru(dpp_3_)]Cl_2_ was utilized to reflect the hypoxia level of 4T1 after different treatments. Firstly, the hypoxia improvement of 4T1 treated with different concentrations of free Met (100, 200, or 500 μg mL^−1^) was quantitatively verified by flow cytometry. Subsequently, the effect of PM@Mn and its controls on the hypoxia level of 4T1 was qualitatively observed using a live cell workstation. Specifically, 4T1 was seeded in a 24‐well plate (8 × 10^4^ cells per well) and cultured overnight. Afterwards, the medium was replaced with fresh medium containing H_2_O_2_ (100 μM) and different preparations (PDA, Met, PM, P@M or PM@Mn, 200 μg mL^−1^ quantified by PDA) and incubated for 24 h. After washing with PBS and staining with Hoechst 33 342, the cells were photographed by a live cell workstation. For qualitative analysis, the above co‐incubated cells were digested and collected, and then detected by flow cytometry.

##### Evaluation of Oxygenation Persistence in Vitro

Oxygen indicating probe [Ru(dpp_3_)]Cl_2_ was utilized to reflect the hypoxia level of 4T1 at different time points. Specifically, 4T1 was seeded in a 24‐well plate (8 × 10^4^ cells per well) and cultured overnight. Afterwards, the medium was replaced with PM@Mn (200 μg mL^−1^) containing H_2_O_2_ (100 μM). Then washing with PBS and staining with [Ru(dpp_3_)]Cl_2_ (20 μM) for 30 min at different time points (0, 2, 4, 8, 16, and 24 h), the cells were digested and collected, and then detected by flow cytometry.

##### Detection of Hypoxic‐Adenosinergic Axis in Vitro

The expressions of HIF‐1α, CD39, and CD73, key indicators of adenosine metabolism, were quantified by flow cytometry after co‐incubation of PM@Mn or its control groups with 4T1. Specifically, 4T1 was seeded in a 12‐well plate (1.5 × 10^5^ cells per well) or a 24‐well plate (1 × 10^5^ cells per well) and cultured overnight. Subsequently, the medium was replaced with fresh medium containing H_2_O_2_ (100 μM) and different preparations (PDA, Met, PM, P@M, or PM@Mn, 200 μg mL^−1^ quantified by PDA), and incubated for 24 h. After washing with PBS, they were digested, collected, and incubated with corresponding dye‐conjugated antibodies, and finally detected by flow cytometry.

##### Western Blot Study

The 4T1 cells were seeded in the 6‐well plates cultured for 24 h, and then treated with different preparations (PDA, Met, PM, P@M or PM@Mn, 200 μg mL^−1^ quantified by PDA) for 24 h. The cells were subjected to a standard western blotting assay to detect the protein expression of HIF‐α and β‐tubulin. The procedures for western blotting assay were briefly described as follows. The total proteins were extracted using a lysate buffer containing protease inhibitor cocktail and quantified by bicinchoninic acid assay. The samples with a same protein concentration were loaded onto the 12% SDS‐PAGE gels, running for 30 min at 80 V and then 90 min at 100 V. The semi‐dry transferring to a membrane was conducted at 12 V for 120 min. The membranes were blocked with tris buffered saline tween (TBST) buffer containing 5% BSA. The primary antibody incubation was performed at 4 °C for overnight at a dilution of 1:1000 for the antibodies to the targeted proteins and 1:20 000 for the housekeeping proteins. After washing for 5 times with TBST (each for 10 min), the membranes were incubated with HRP‐labeled Goat Anti‐Mouse IgG (H + L) (1:1000 dilution, Beyotime, China) for 45 min at room temperature. After thorough wash and a standard staining process, the membranes were exposed to a digital imager.

##### Evaluation of Photothermal Killing Effect

CCK‐8 and apoptosis assay kit were utilized to investigate the killing effect of PM@Mn on 4T1 cells at different irradiation temperatures. The cell viability of 4T1 treated with different irradiation temperatures (37, 39, 42, or 45 °C) was measured by the CCK‐8 method, and the detailed steps referred to Cytotoxicity Evaluation in Vitro. Moreover, Annexin V/PI apoptosis assay kit was used to detect the apoptosis and necrosis of 4T1 treated with different irradiation temperatures. 4T1 was seeded in a 24‐well plate (8 × 10^4^ cells per well) and cultured overnight. Afterwards, the medium was replaced with fresh medium with or without PM@Mn (200 μg mL^−1^), and incubated for 4 h. The cells were irradiated with 808 nm laser at different temperatures (39, 42, or 45 °C) for 8 min, and incubated for 12 h. After washing with PBS, they were digested, collected, stained according to the instructions, and finally detected by flow cytometry.

##### Evaluation of Photothermal ICD Induction

The expression of ICD markers CRT and HMGB1 was detected to evaluate the induction of ICD in tumor cells treated with PM@Mn under different irradiation temperatures. For the detection of CRT, 4T1 were seeded in 24‐well plates (8 × 10^4^ cells per well) and cultured overnight. Afterwards, the medium was replaced with fresh medium with or without PM@Mn (200 μg mL^−1^) and incubated for 4 h. The above cells were irradiated with 808 nm laser at specific temperatures (39, 42, or 45 °C) for 8 min, and incubated for 12 h. After being fixed with paraformaldehyde, washed with PBS, and blocked with 5% FBS, they were incubated with CRT antibody overnight. Subsequently, Alexa fluor 488‐labeled secondary antibody was added after washing with PBS, and incubated for 1 h. Finally, a live cell workstation was employed to shoot Hoechst 33 342‐stained cells. As for the qualitative analysis, the above cells were digested, collected, and detected by flow cytometry.

For HMGB1, the steps of cell inoculation, material addition, irradiation, fixation and washing are the same as those for CRT detection described above. Then, after permeabilization with 0.5% Triton X‐100, washed with PBS and blocked with 5% FBS, they were incubated with Alexa fluor 488‐HMGB1 antibody for 12 h. Ultimately, cells stained with Hoechst 33 342 were photographed.

##### Detection of ATP and Adenosine Content

The kit was utilized to quantitatively detect the contents of ATP and adenosine in 4T1 cells of different treatment groups to investigate the effect of PM@Mn and its control groups on ATP‐based adenosine metabolism. Specifically, 4T1 was seeded in a 24‐well plate (8 × 10^4^ cells per well) and cultured overnight. Afterwards, the medium was displaced with fresh medium containing PDA or PM@Mn (200 μg mL^−1^), and incubated for 4 h. The cells were irradiated with 808 nm laser at 45 °C for 8 min, and the incubation was continued. The supernatants of each group were collected before irradiation and after irradiation 2, 4, 8, and 12 h, and then quantitatively detected with enhanced ATP assay kit and adenosine assay kit, respectively.

##### Evaluation of BMDCs Maturation

First, bone marrow‐derived dendritic cells (BMDCs) were obtained by the modified Inaba method, and the toxicity of PM@Mn to BMDCs was detected by the CCK‐8. Subsequently, co‐incubation experiments were designed using Transwell system, and the levels of BMDCs maturation in different treatment groups were analyzed by flow cytometry and Elisa. Specifically, immature BMDCs were seeded in 24‐well plates (8 × 10^5^ cells per well) and cultured overnight. Meanwhile, 4T1 cells were seeded in the upper chamber of the Transwell system (2 × 10^5^ cells per well). Afterwards, the medium in the Transwell system was substituted with fresh medium containing PDA or PM@Mn (200 μg mL^−1^) and H_2_O_2_ (100 μM), and incubated for 4 h. The cells were irradiated with 808 nm laser at 45 °C for 8 min, and then all Transwell systems inoculated with 4T1 were transferred to the corresponding well plates inoculated with BMDCs, and co‐incubated for 24 h. Among them, untreated BMDCs were used as negative control, LPS (1 μg mL^−1^) treated group was used as a positive control. The supernatant of the lower chamber of the co‐incubation system was collected, and then the secretion of TNF‐α was detected with an Elisa kit according to the instructions. Additionally, the above BMDCs were collected, incubated with dye conjugated antibodies (APC‐CD11c, PE‐CD86, and FITC‐MHCII), and detected by flow cytometry.

##### Evaluation of Oxygenation Persistence in Vivo

4T1 cells (5 × 10^5^ cells per mouse) were subcutaneously injected into the right bottom breast pad of BABL/c female mice. When the tumor grew to 75≈100 mm^3^, the mice were randomly divided into 6 groups (0, 2, 4, 8, 16, and 24 h, *n* = 3). All mice were intravenously injected with 200 μL PM@Mn (PDA: 20 mg kg^−1^). Mice were euthanized at different time points (0, 2, 4, 8, 16, and 24 h), and tumors tissues were dissected. Part of the tumor tissue obtained at the treatment endpoint was utilized to prepare single‐cell suspension which was then stained with [Ru(dpp_3_)]Cl_2_ (20 μM) for 30 min and subjected to flow cytometry test.

##### Evaluation of Antitumor Effect in Vivo

4T1 cells (5 × 10^5^ cells per mouse) were subcutaneously injected into the right bottom breast pad of BABL/c female mice. When the tumor grew to 75≈100 mm^3^, the mice were randomly divided into 5 groups (PBS, PDA, PM@Mn, PDA + L, and PM@Mn + L, *n* = 5). All mice were intravenously injected with 200 μL preparation of the corresponding components (PDA: 20 mg kg^−1^, Met: 3 mg kg^−1^) every 3 days.^[^ ^25^ ^]^ After 2 h, laser irradiation (45 °C, 8 min) was performed, and a total of 3 treatments. On day 5 after the last administration, all mice were euthanized, and tumors, lymph nodes, and organ tissues were dissected. During this period, the tumor length (L), width (W) and body weight were measured every other day. The calculation formulas of tumor volume and tumor inhibition rate are as follows:

(3) $$V\left({\text{mm}}^{3}\right)=\frac{L\times W^{2}}{2}$$

(4) $$TumorInhibitionRate\left(\%\right)=\frac{V_{\text{PBS}}-V}{V_{\text{PBS}}}\times 100\%$$

##### Evaluation of Adenosine Metabolism in Tumor Tissue

The effect of PM@Mn on adenosine metabolism under laser irradiation was investigated by detecting the expression of key indicators of adenosine metabolism and the content of adenosine in tumor tissues of each treatment group. Concretely, part of the tumor tissue obtained at the treatment endpoint was utilized to prepare single‐cell suspension for subsequent staining of the corresponding flow cytometry antibody, and part was utilized for subsequent immunofluorescence experiments, thereby jointly evaluating the expression of HIF‐1α, CD39, and CD73. Moreover, the tumors obtained from each group were weighed, cut into pieces, and thoroughly ground with PBS containing protease inhibitors and ENHA (adenosine deaminase inhibitor) at a weight‐to‐volume ratio of 1:5. The supernatant of the above tissue homogenate was collected by centrifugation, and detected according to the instructions of the adenosine assay kit.

##### Analysis of Antitumor Immunity in Vivo

The tumor local immune response in different treatment groups was investigated by analyzing the infiltration of T lymphocytes. Tumor tissue was cut into pieces and enzymatically hydrolyzed (2 mg mL^−1^ collagenase + 2.2 μL mL^−1^ DNase) to obtain single‐cell suspension. After being blocked by Fc‐Block, the single‐cell suspension was incubated with the corresponding flow antibody to investigate the infiltration of CD4^+^ and CD8^+^ T lymphocytes (APC‐CD3, PE/Cy7‐CD8, FITC‐CD4, and PE‐IFN‐γ) and the proportion of regulatory T cells (APC‐CD3, FITC‐CD4, PE/Cy7‐CD25, and PE‐Foxp3). Among them, the staining of PE‐IFN‐γ and PE‐Foxp3 required membrane breaking and nucleation breaking, respectively.

Systemic immune response in different treatment groups was examined by analyzing the maturation of DCs in the lymph nodes and the infiltration of immune cells in the spleen. The lymph node single‐cell suspension obtained by grinding was blocked by Fc‐Block and incubated with corresponding flow antibodies (APC‐CD11c, FITC‐CD80, PE‐CD86, and Brilliant Violet 421 anti‐mouse I‐A/I‐E) to explore the maturation of DCs. The spleen single‐cell suspension obtained by grinding and erythrocyte lysis was blocked by Fc‐Block and incubated with the corresponding flow antibody to detect the infiltration of T lymphocytes (APC‐CD3, FITC‐CD4 and PE/Cy7‐CD8) and MDSCs (PE‐CD45, APC‐CD11b, and PE/Cy7‐Gr‐1) in the spleen.

##### Analysis of Biological Safety in Vivo

In order to investigate the biological safety during the treatment, the main organs (heart, liver, spleen, lung, and kidney) of mice in each group were stained with hematoxylin‐eosin (H&E), and then observed with the microscope. Furthermore, the corresponding kits were utilized to detect the levels of ALT, AST, and CRE in the serum through orbital blood sampling.

##### Statistical Analysis

FlowJo 7.6 or FlowJo 10 was employed to process flow data, and GraphPad Prism 8.4.3 was utilized for data analysis. The data of each group was presented as mean ± standard deviation (± SD), and “n” represented the number of samples within a group. One‐way ANOVA were used for multiple comparisons when more than two groups were compared, and Student's *t*‐test was used for two‐group comparisons. Among them, “ns” indicated no statistical difference, “*” indicated statistical difference, where *P < 0.5, **P < 0.1, ***P < 0.01, ****P < 0.001.

## Conflict of Interest

The authors declare no conflict of interest.

## Supporting information

Supplementary Material

## Data Availability

The data that support the findings of this study are openly available in GJJ at https://doi.org/10.1002/smsc.202300242, reference number 456.
