# Supplementary material for: Suppressing the Hypoxia‐Adenosinergic Axis by a Tailored Nanoreactor for Enhanced Photothermal Immunotherapy
Source: Small Sci. 2024 Feb 5;4(4):2300242. doi: 10.1002/smsc.202300242 (PMC11935205; doi:10.1002/smsc.202300242)
Supplement: Supplementary file 1 — Supplementary Material [file SMSC-4-2300242-s001.pdf]

Supporting Information

Suppressing the Hypoxia-Adenosinergic Axis by a Tailored Nanoreactor for Enhanced Photothermal Immunotherapy

Jingjing Gu [a,1], *Jiao Chang* [b,1], *Shiyu Chen* [a], *Hui Zhi* [a], *Jiuyuan Sun* [b], *Weimin Yin* [a], *Tingting Zhang* [b], *Jie Zang* [b], *Yuge Zhao* [b], *Yiqiong Liu* [b], *Xiao Zheng* [b], *Leiyu Feng* [c], *Yongyong Li* [b,*], *Haiqing Dong* [a,*]

[a] Key Laboratory of Spine and Spinal Cord Injury Repair and Regeneration, Ministry of Education, Tongji Hospital, The Institute for Biomedical Engineering & Nano Science, School of Medicine, Tongji University, Shanghai 200092, P. R. China.

[b] Shanghai Skin Disease Hospital, The Institute for Biomedical Engineering & Nano Science, School of Medicine, Tongji University, Shanghai 200092, P. R. China.

[c] State Key Laboratory of Pollution Control and Resources Reuse, School of Environmental Science and Engineering, Tongji University, Shanghai 200092, P. R. China

* Corresponding Authors

E-mail: inano_donghq@tongji.edu.cn (Haiqing Dong)

yongyong_li@tongji.edu.cn (Yongyong Li)

[1] Jingjing Gu and Jiao Chang contributed equally to this work.

1. Experimental Section

Materials: Dopamine hydrochloride ($C_8H_{12}ClNO_2$, DA·HCl), lipopolysaccharide (LPS) and erythro-9-(2-hydroxy-3-nonyl) adenine hydrochloride ($C_{14}H_{24}ClN_5O$, EHNA) were purchased from Sigma-Aldrich Shanghai Trading Co., Ltd. Potassium permanganate ($KMnO_4$) was obtained from Hebei Wuluo Pharmaceutical Co., Ltd. Metformin hydrochloride (C_4HClN_5, Met·HCl) and hydrogen peroxide (H_2O_2, 30 wt.%) were purchased from Sinopharm Chemical Reagent Co., Ltd. Tris (4,7-diphenyl-1,10-phenanthroline) ruthenium(II) dichloride ($[Ru(dpp)_3]Cl_2$), rhodamine B isothiocyanate, ammonia ($NH_3·H_2O$, 28~30 wt.%) and anhydrous ethanol were purchased from Shanghai Aladdin Biochemical Technology Co., Ltd. CCK-8 cytotoxicity assay kit, Annexin V/PI assay kit and enhanced ATP assay kit were purchased from Beyotime Biotech Co., Ltd. The mouse adenosine assay kit was bought from Shanghai Jingkang Biological Engineering Co., Ltd.

The following antibodies were used: calreticulin rabbit monoclonal antibody (Beyotime), Alexa Fluor 488 anti-mouse HMGB1 (Dakewe), rabbit anti-HIF-1α antibody (Bioss), rabbit anti-CD39 antibody (Bioss), rabbit anti-CD73 antibody (Bioss), the purified anti-mouse CD16/32 (Biolegend), PE anti-mouse HIF-1α (eBioscience), PE anti-mouse CD39 (Biolegend), APC anti-mouse CD73 (Biolegend), APC anti-mouse CD3 (Biolegend), PE/Cy7 anti-mouse CD8 (Biolegend), FITC anti-mouse CD4 (Biolegend), PE anti-mouse IFN-γ (Biolegend), PE/Cy7 anti-mouse CD25 (Biolegend), PE anti-mouse Foxp3 (Biolegend), APC anti-mouse CD11b (Biolegend), PE anti-mouse CD45 (Biolegend), PE/Cy7 anti-mouse Gr-1 (Biolegend), APC anti-mouse CD11c (eBioscience), FITC anti-mouse CD80 (Biolegend), PE anti-mouse CD86 (eBioscience) and Brilliant Violet 421™ anti-mouse MHCII (Biolegend).

Cell Culture and Animals: Both the human kidney epithelial cell line 293T and the mouse breast cancer cell line 4T1 were obtained from the Shanghai Cell Bank of the Chinese Academy of Sciences, and were subcultured in DEME and RPMI-1640 complete media supplemented with 10% fetal bovine serum (FBS) and 1% penicillin-streptomycin, respectively. Bone marrow-derived dendritic cells (BMDCs) were extracted from the bone marrow of BALB/c mice by the modified Inaba method, and treated with the addition of cytokines (40 ng mL^{-1} GM-CSF and 20 ng mL^{-1} IL-4) RPMI 1640 complete medium for induction.

Experimental animals BALB/c female mice, aged 5~7 weeks, were purchased from Shanghai Slack Experimental Animal Co., Ltd., and bred in the specific pathogen-free (SPF) animal room of Tongji University Animal Center. All animal experiments in this project were approved by

the Animal Experiment Ethics Committee of Tongji University (Animal Ethics: TJAA07720101).

2. Supporting Figures

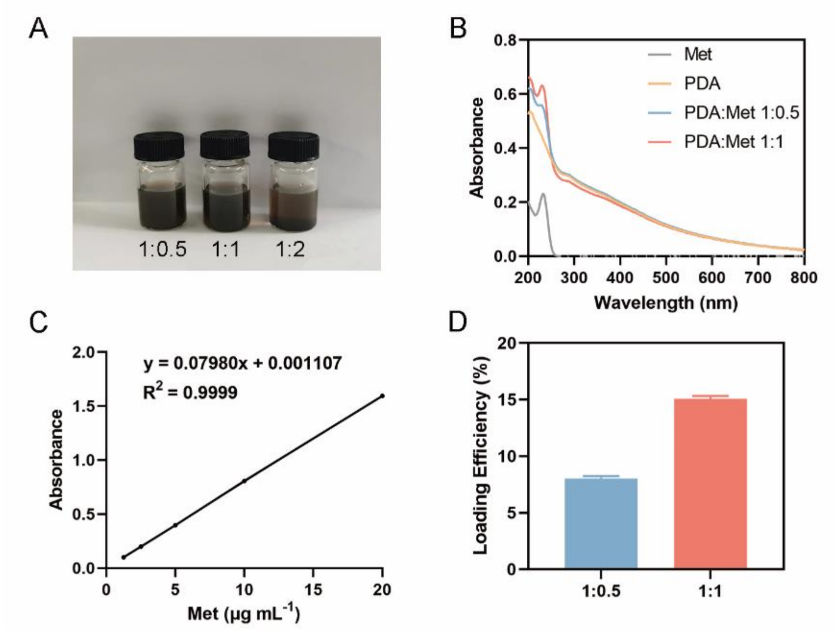

Figure S1. (A) Photos of PM nanoparticles with different feeding mass ratio. (B) Ultraviolet-visible absorption spectra of nanoparticles with different components. (C) The standard curve of Met. (D) Drug loading efficiency of PM nanoparticles with different feeding mass ratio. The data are presented as the means ± SD (n = 3).

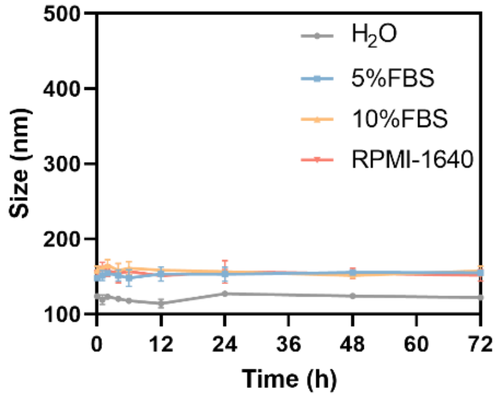

Figure S2. Particle size variations of PDA in different dispersion media (H_2O, 5%FBS, 10%FBS and RPMI-1640 complete medium). The data are presented as the means ± SD (n = 3).

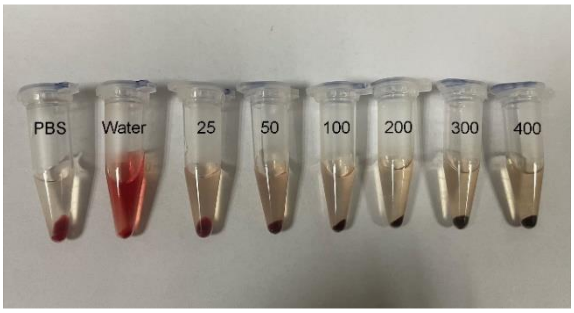

Figure S3. Pictures of PM@Mn hemolysis at different concentrations (0, 25, 50, 100, 200, 300 and 400 μg mL^{-1}).

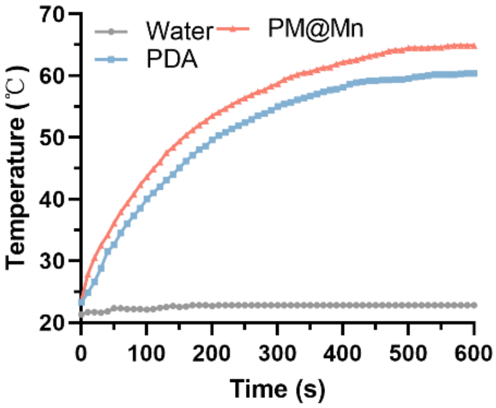

Figure S4. Temperature curves of PDA and PM@Mn (200 μg mL^{-1}) under 808 nm laser irradiation (0.75 W cm^{-2}).

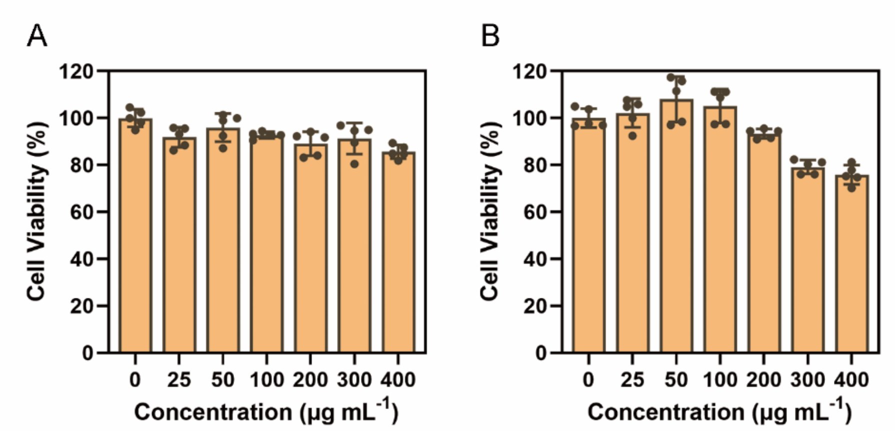

Figure S5. The cytotoxicity of PM@Mn (0, 25, 50, 100, 200, 300 and 400 μg mL^{-1}) to (A) 293T and (B) 4T1. The data are presented as the means ± SD (n = 5).

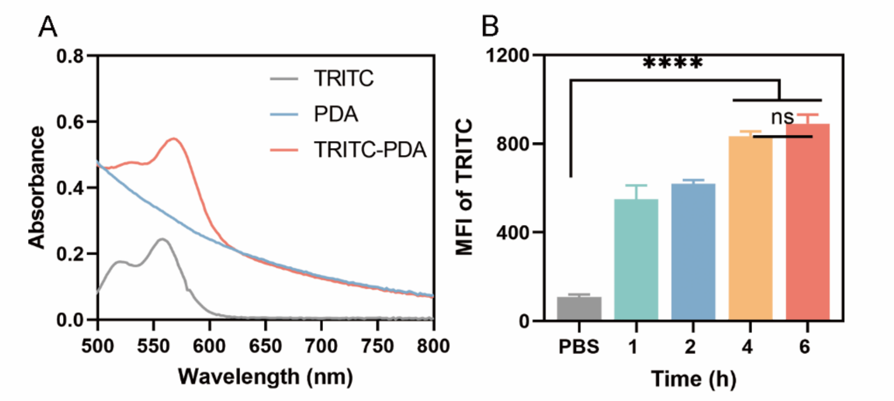

Figure S6. (A) Ultraviolet-visible absorption spectra of TRITC-PDA and its control groups. (B) The MFI of TRITC-PM@Mn and 4T1 after different incubation times (1, 2, 4 and 6 h). The data are presented as the means ± SD (n = 3). (**** $p < 0.0001$).

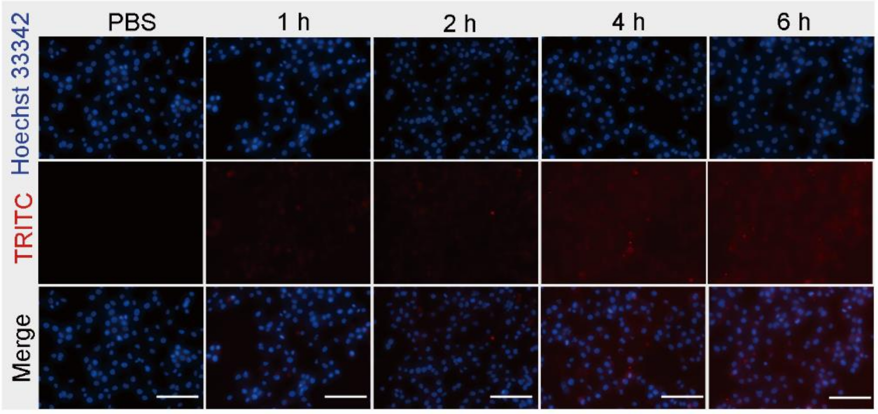

Figure S7. Images of TRITC-PM@Mn and 4T1 incubated at different times taken by living cell workstation (scale bar: 100 μm).

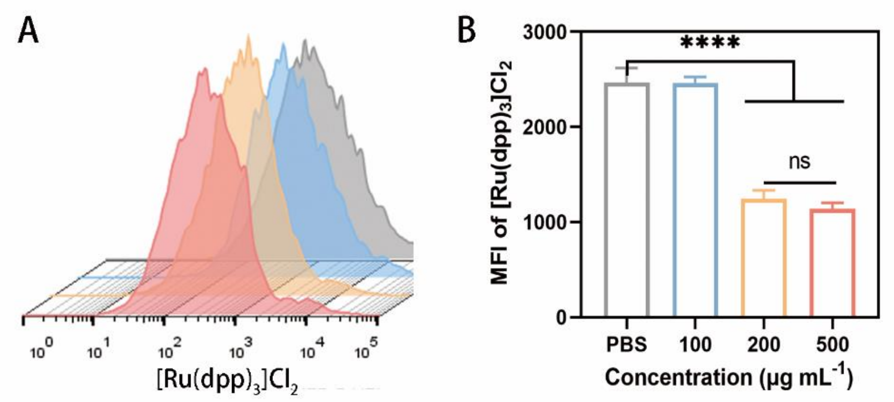

Figure S8. (A) Representative flow cytometry histogram and (B) MFI quantitative analysis of $[Ru(dpp_3)]Cl_2$ fluorescence after different concentrations of free Met co-incubated with 4T1. The data are presented as the means ± SD (n = 3). (ns, not significant and **** $p < 0.0001$).

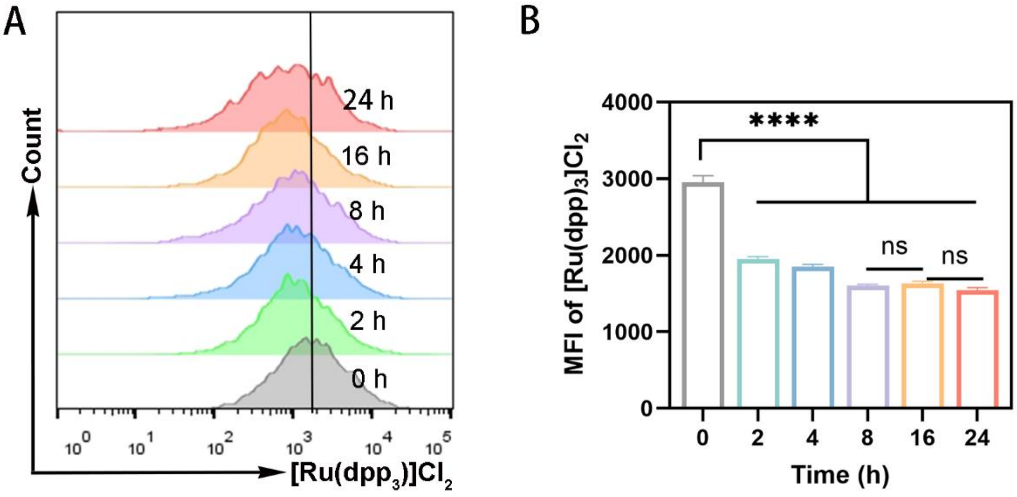

Figure S9. (A) Representative flow cytometry histogram and (B) MFI quantitative analysis of $[Ru(dpp_3)]Cl_2$ fluorescence after different incubation times with PM@Mn and 4T1 (0, 2, 4, 8, 16 and 24 h). The data are presented as the means ± SD (n = 3). (ns, not significant and **** *p* < 0.0001).

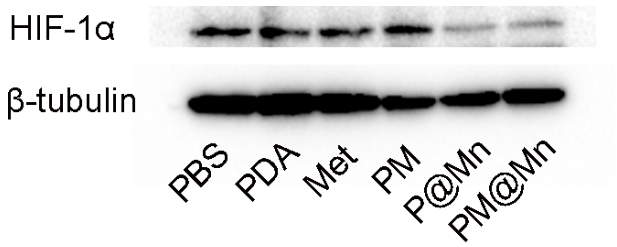

Figure S10. Western Blot analysis of HIF-1α expression levels in PM@Mn and its control groups (PBS, PDA, Met, PM and P@Mn, 200 μg mL^{-1} calculated by PDA) after incubation with 4T1. The gels were run in parallel.

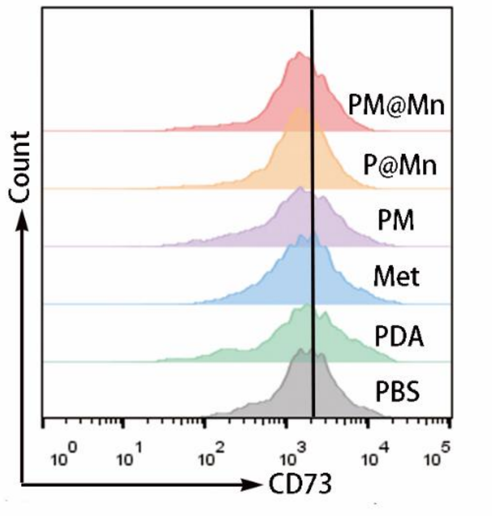

Figure S11. Representative flow cytometry histogram of APC-CD73 in PM@Mn and its control groups (200 μg mL^{-1} calculated by PDA; Met calculated at 15% loading rate) after incubation with 4T1.

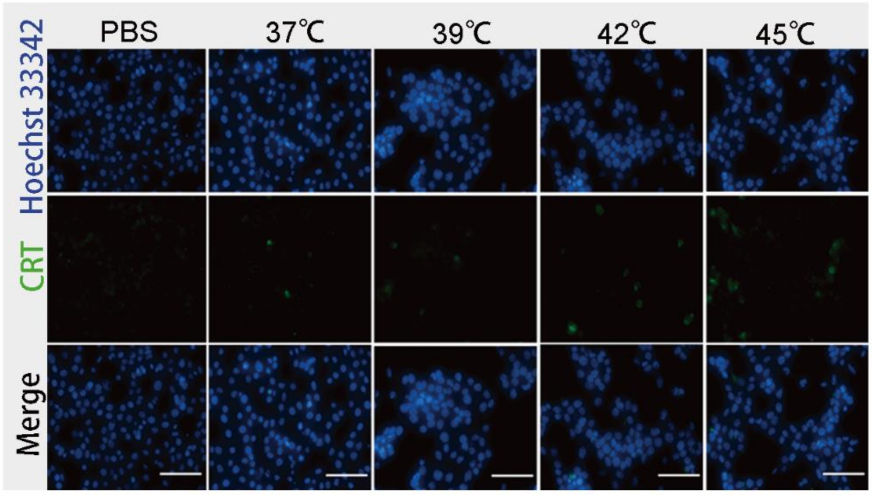

Figure S12. CRT expression in 4T1 under different irradiation temperatures observed by living cell workstation (scale bar: 100 μm).

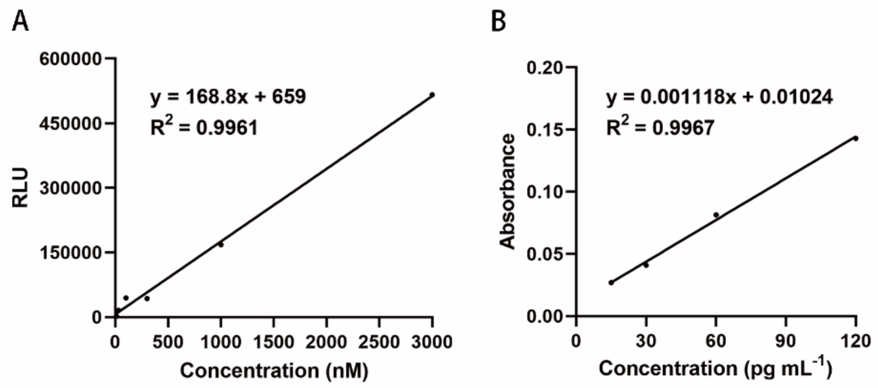

Figure S13. (A) The standard curve of ATP via enhanced ATP assay kit. (B) The standard curve of adenosine via mouse adenosine assay kit.

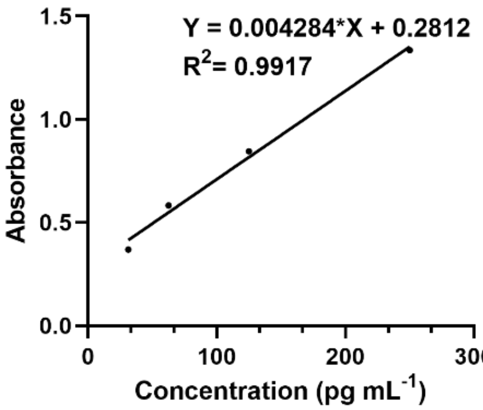

Figure S14. The standard curve of TNF-α via mouse adenosine assay kit.

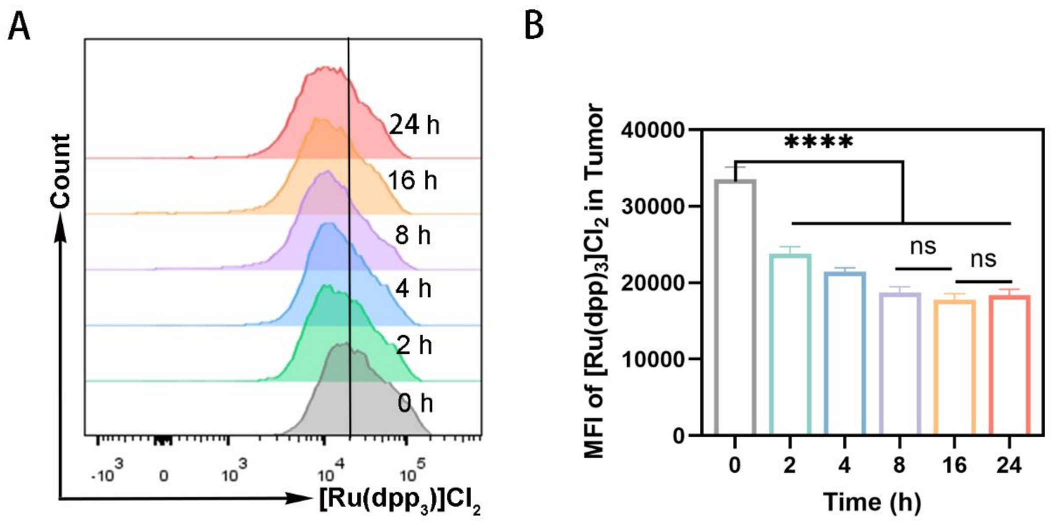

Figure S15. (A) Representative flow cytometry histogram and (B) MFI quantitative analysis of $[Ru(dpp_3)]Cl_2$ fluorescence in whole-cell extracts from 4T1 tumor tissues after injection PM@Mn for different times (0, 2, 4, 8, 16 and 24 h). The data are presented as the means ± SD (n = 3). (ns, not significant and **** *p* < 0.0001).

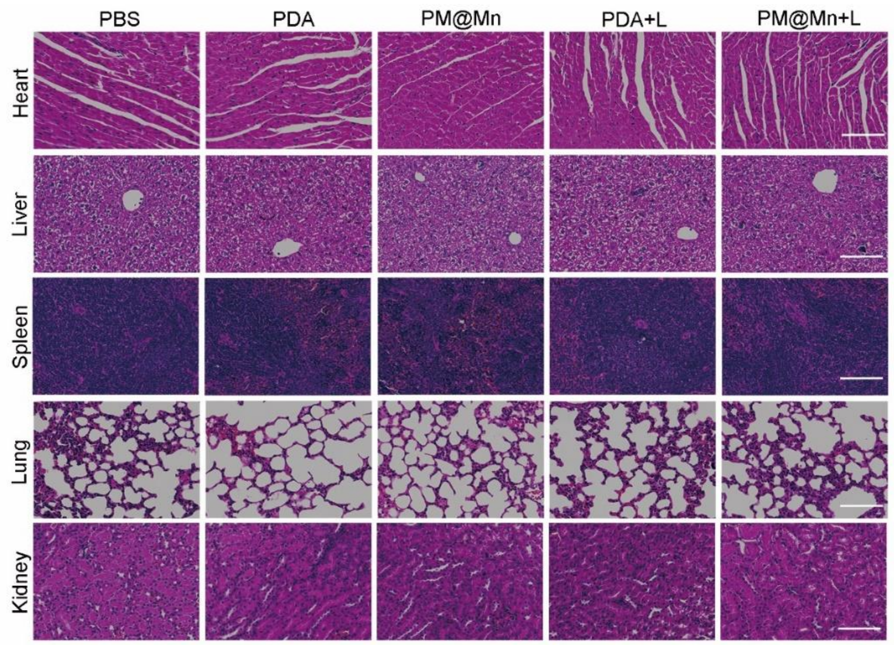

Figure S16. H&E staining images of organs in different treatment groups after treatment (scale bar: 100 μm).

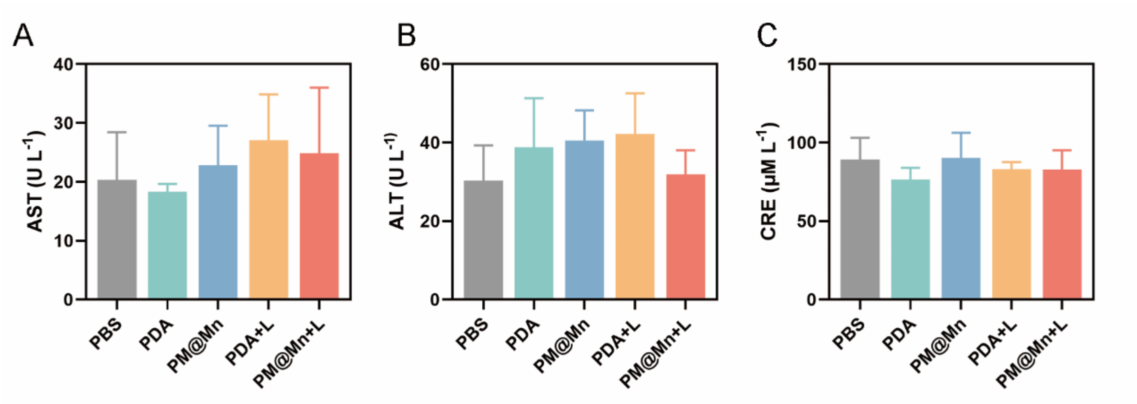

Figure S17. Contents of (A) AST, (B) ALT and (C) CRE in the serum of different treatment groups after treatment. The data are presented as the means ± SD (n = 5).

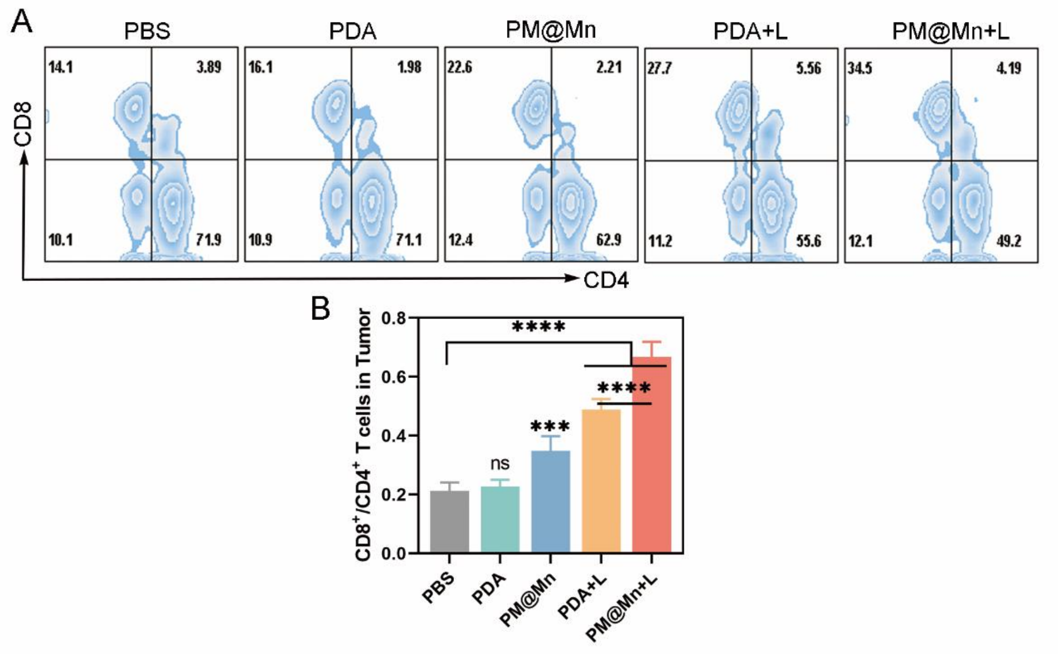

Figure S18. Representative flow cytometry plots of the proportion of CD8$^+$ T cells and CD4$^+$ T cells (gated on CD3$^+$ T cells) infiltration in tumor tissues of different treatment groups and c) the analysis of CD8$^+$/CD4$^+$ T cells ratio. The data are presented as the means ± SD (n = 5). (ns, not significant, *** p < 0.001 and **** p < 0.0001).

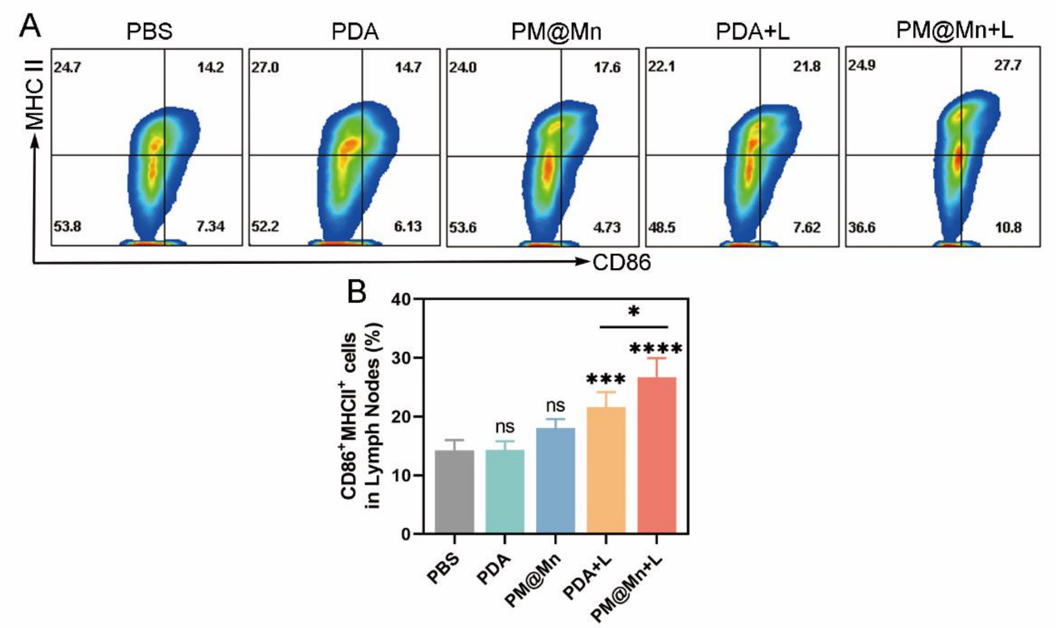

Figure S19. (A) Representative flow cytometry plots and (B) the percentages of $CD86^{+}MHC$ II^{+} DCs (gated on $CD11c^{+}$ cells) infiltration in lymph nodes of different treatment groups. The data are presented as the means ± SD (n = 5). (ns, not significant, * $p < 0.05$, *** $p < 0.001$ and **** $p < 0.0001$).

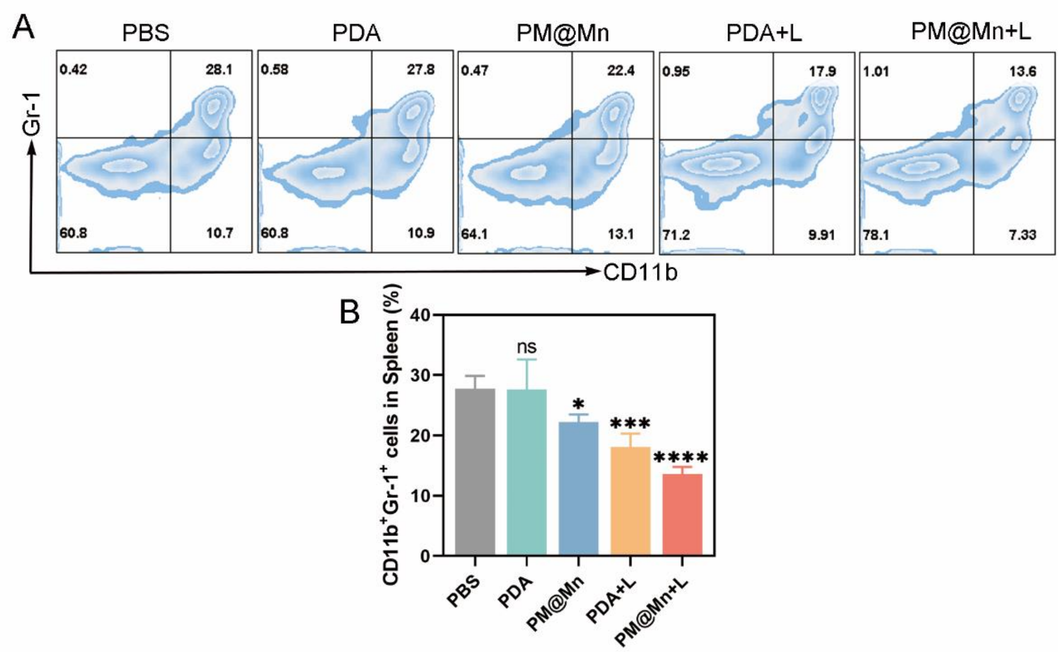

Figure S20. (A) Representative flow cytometry plots and (B) the percentages of $CD11b^{+}Gr\text{-}1^{+}$ cells (gated on $CD45^{+}$ cells) infiltration in the spleen of different treatment groups. The data are presented as the means ± SD (n = 5). (ns, not significant, * $p < 0.05$, *** $p < 0.001$ and **** $p < 0.0001$).

s
